# Automated flight-interception traps for interval sampling of insects

**DOI:** 10.1371/journal.pone.0229476

**Published:** 2020-07-10

**Authors:** Janine Bolliger, Marco Collet, Michael Hohl, Martin K. Obrist

**Affiliations:** 1 WSL, Swiss Federal Research Institute, Birmensdorf, Switzerland; 2 WSL Institute for Snow and Avalanche Research SLF, Davos, Switzerland; Durham University, UNITED KINGDOM

## Abstract

Recent debates on insect decline require sound assessments on the relative drivers that may negatively impact insect populations. Often, baseline data rely on insect monitorings that integrate catches over long time periods. If, however, effects of time-critical environmental factors (e.g., light pollution) are of interest, higher temporal resolution of insect data is required during very specific time intervals (e.g., between dusk and dawn). Conventional time-critical insect trapping is labour-intensive (manual activation/deactivation) and temporally inaccurate as not all traps can be serviced synchronically at different sites. Also, temporal shifts of environmental conditions (e.g., sunset/sunrise) are not accounted for. We present a battery-driven automated insect flight-interception trap which samples insects during seven user-defined time intervals. A commercially available flight-interception trap is fitted to a turntable containing eight positions, seven of them holding cups and one consisting of a pass-through hole. While the cups sample insects during period of interest, the pass-through hole avoids unwanted sampling during time-intervals not of interest. Comparisons between two manual and two automated traps during 71 nights in 2018 showed no difference in caught insects. A study using 20 automated traps during 104 nights in 2019 proved that the automated flight-interception traps are reliable. The automated trap opens new research and application possibilities as arbitrary insect-sampling intervals can be defined. The trap proves efficient, saving manpower and associated costs as activation/deactivation is required only every seven sampling intervals. In addition, the timing of the traps is accurate, as all traps sample at exactly the same intervals and ensure comparability. The automated trap is low maintenance and robust due to straightforward technical design. It can be controlled manually or via smartphone through a Bluetooth connection. Full construction details are given in Appendices.

## Introduction

Passive traps are widely applied in documenting and monitoring insect biodiversity [1, 2]. Among traps, transparent flight-interception traps have been successfully used for many flying insect orders [3–7]. For most trap applications such as biodiversity monitoring, insect sampling does not require a high temporal resolution and traps are put in place to run for longer time periods, typically days to weeks [6]. However, in the context of large-scale insect decline, knowledge on how individual environmental drivers act on the populations are needed [8, 9]. One such driver, artificial light at night and its mostly negative impacts on insects, has triggered interest among researchers in sampling during defined time intervals (i.e., nights) [10, 11]. While such sampling is well established for other organisms, e.g. recording acoustic signals of bats [12], we are not aware of time-interval sampling devices for insects. To date, two trap visits per defined time interval are required: trap activation at the beginning of the sampling interval and deactivation and collection of the insects at the end of the time interval. Apart from being labour-intensive, such manual sampling of time intervals is inherently inaccurate with respect to sampling synchrony because handling the traps takes time and in general experiments consist of numerous traps at different, often distant sites. In addition, seasonal time shifts of narrow time intervals such as sunset or sunrise cannot be accounted for. Lastly, manually operated traps may results in lower sample sizes than desired because manpower is limited during weekends and public holidays.

We present an extended automated flight-interception trap to catch insects, whose design, construction and effectiveness allows for sampling during user-defined time intervals. While a standard commercially available flight-interception trap is used to ensure comparable trappability, the trap is supplemented by an automated device, providing temporal fractionation of captures and extending on the storage capacity of the sampled insects. The new, automated sampling device (1) allows for any user-defined sampling intervals; (2) is very efficient as site visits to empty and re-activate the traps are required only after seven consecutive sampling intervals (e.g., seven nights); (3) allows for accurate and synchronized exposure times of each of the seven sampling cups in every trap used; (4) is reliable and robust due to straightforward mechanical and electronic design and controllable via smartphone through a Bluetooth connection.

## Material, methods and results of trap evaluations

### Trap design

**Fig 1:**
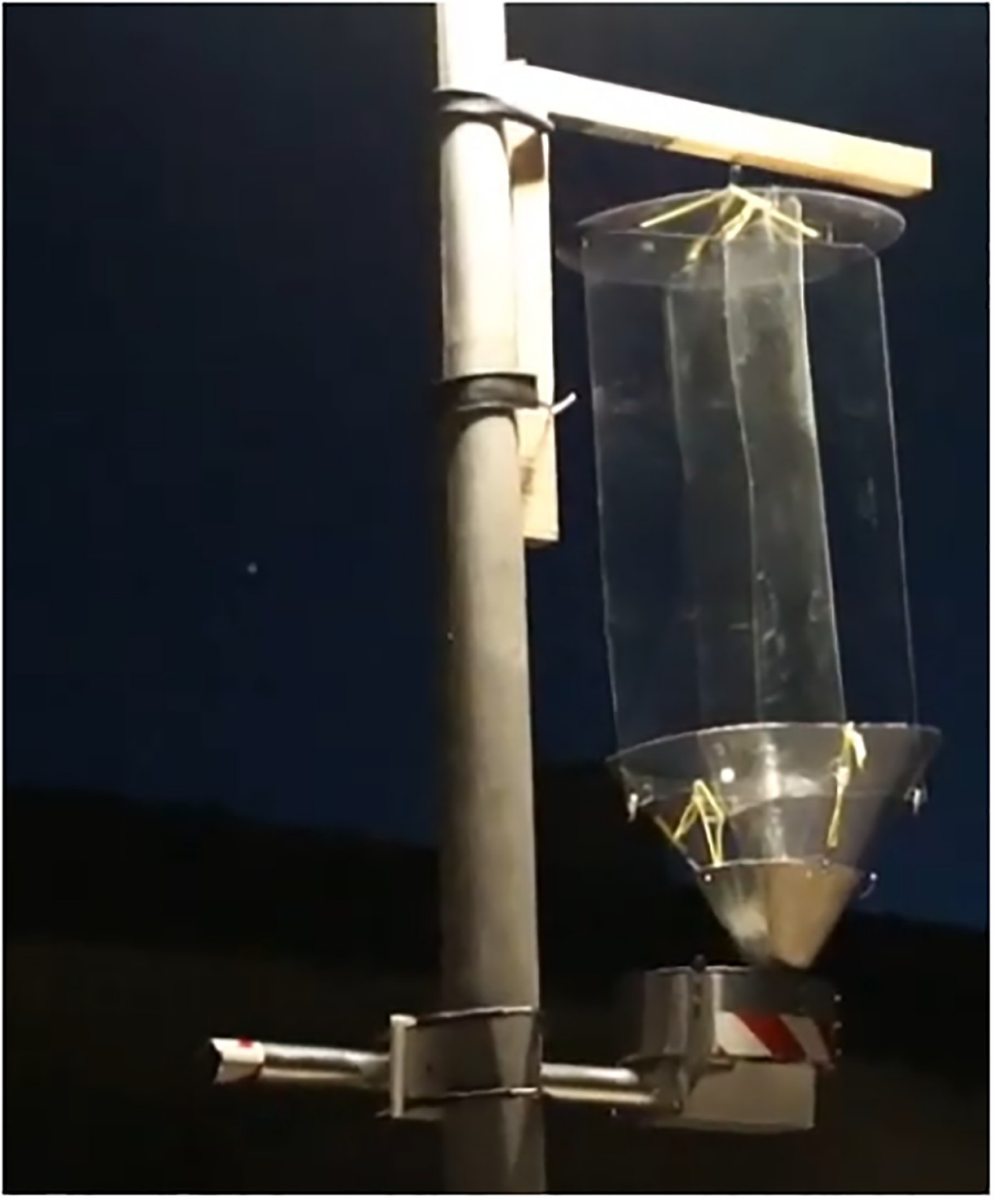
The automated insect trap mounted on a street-light pole. While the commercially available transparent flight-interception trap remains unmodified, the insect sampling unit is optimized: a turntable containing seven cups is rotated by a stepper motor fed by a rechargeable battery. The seven cups contain a liquid (e.g. water with/without biocide) and collect insects during seven user-defined time intervals (e.g., nights).

**Fig 2:**
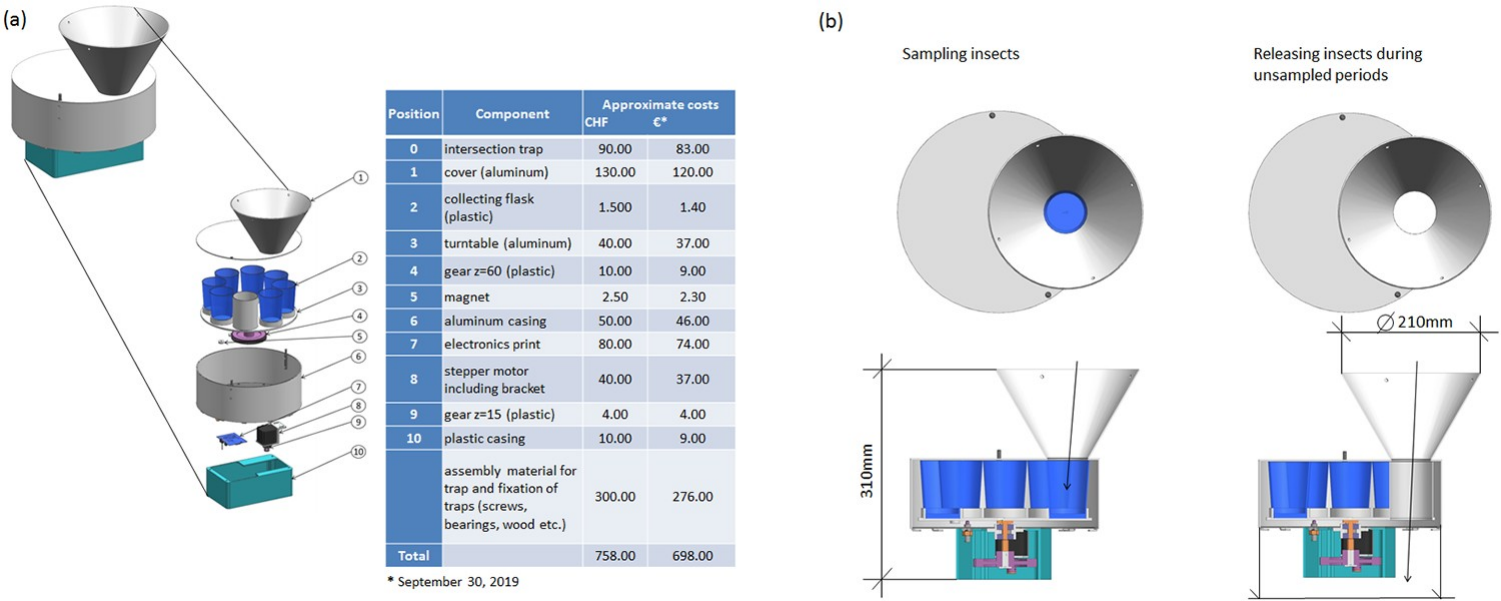
**(a)** Design and components of the turntable to optimize insect sampling including a list of required components and their approximate costs. Labor costs are not included; **(b)** position during periods of sampling (cups placed underneath the trap funnel) and non-sampling periods, when insects are released through the pass-through hole.

Our automated insect trap relies on a commercial flight-interception trap (Polytrap^®^ https://cahurel-entomologie.com/shop/pieges/434-piege-polytrap.html) whose transparent and light plastic construction consists of two crossed, clear PET sheets which represent an omnidirectional barrier for flying insects, a trap roof, and a funnel and beaker filled with liquid to collect the insects (Fig 1). These traps are broadly applied in entomological research, e.g., [5, 6]. While the trappability of the commercial insect trap remains the same, the actual insect sampling is automated and optimized in the new trap: a turntable holding cups to trap insects during user-defined time intervals is rotated by a stepper motor located underneath the actual flight-intersection trap (Figs 1 and 2). The motor is fed by a rechargeable battery. The exact duration of the user-defined time intervals are stored electronically in a lookup-table contained in the firmware (S1 Appendix for technical details). The turntable contains seven cups to trap insects—one cup for each time interval (Fig 2a). The cups may contain water, ideally mixed with a light biocidal solvent (e.g., Rocima GT, Acima AG, CH-9471 Buchs / Rohm and Haas Co.) to facilitate sinking of insects and prevent rotting of the fluid in warm weather. Alternatively alcohol solutions could be used to allow for further analyses (e.g. DNA) of the insects, but we have not tested the evaporation loss of such solutions over time in the traps. In addition to the seven cups, the turntable contains a pass-through hole (Fig 2b). The turntable rotates in such a way, that the hole’s position moves under the trap during intervals where insects are not trapped. This ensures that any insect falling into the funnel during non-trapping times is immediately released through the hole (Fig 2b; see also https://www.youtube.com/watch?v=aGFRxYjO1rM). At the beginning of the first user-defined time interval, the first cup (Pos. 1) is positioned underneath the funnel to catch insects. At the end of the sampling time interval, the turntable positions the pass-through opening under the funnel so that insects are no longer sampled (Fig 2). The exact position of each cup and the pass-through hole are determined using two magnets and a Hall-effect sensor built into the print (S1 Appendix for technical details). The insect trap can be controlled through a smartphone application connected via Bluetooth. For this the APP ‘BGX Commander’ is required on the smartphone (S1 Appendix). Details on the electronic scheme and the state chart of the electronic software are shown in S2 and S3 Appendices. Firmware for the electronic controller, materials needed to build all electronics and the layout for the printed circuit board are all provided in S4–S6 Appendices respectively. Finally, mechanical construction plans can be found in S7 Appendix.

The automated insect trap can be dimensioned differently to contain more or less cups than seven. Our design was largely driven by the specific research questions in mind, namely night-time insect catching during a full week. Sampling seven time intervals thus represented a trade-off between trap and cup size, trap weight (3.6 kg) and servicing intervals. As the traps are serviced only once a week, the cups had to be large enough to take up enough water in order to counteract evaporation during hot weather periods.

### Trap evaluation

**Fig 3:**
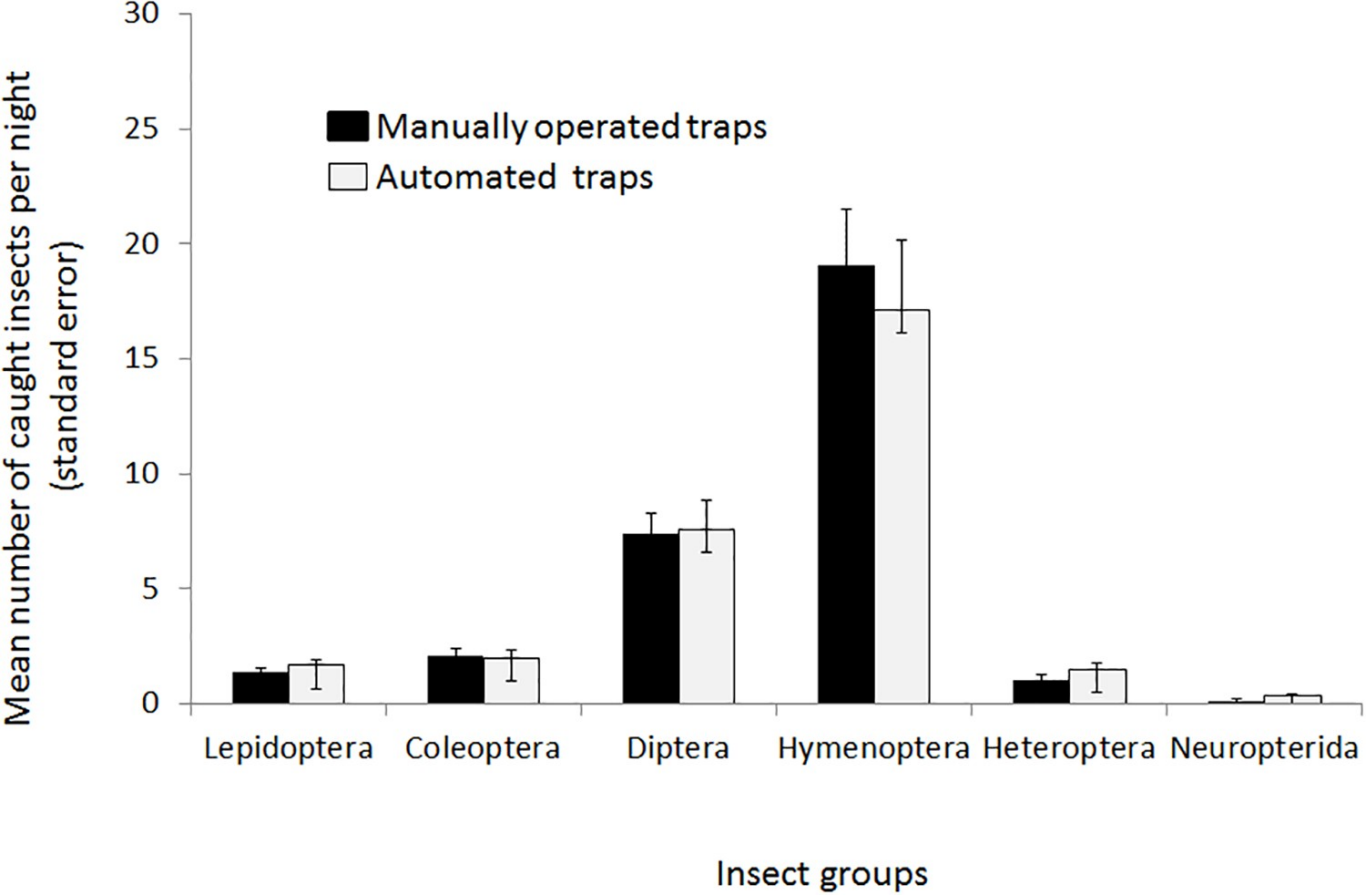
Comparison of the mean number of caught insects for automated and manually operated insect traps during 71 nights (data from summer 2018) for two traps.

We compared the automated insect interval trap with the original manually operated trap to test the feasibility of the trap’s design and the reliability of the electronical and mechanical components. In a pilot study, insects were caught at street light poles approx. 30 m apart from each other during 71 nights between June and mid-July 2018 in the town of Birmensdorf (ZH), Switzerland, using two automated insect traps and two manually operated traps. No permit was needed to catch insects, and as EKZ owned the street lights also no access permission was required. The caught insects were sorted into six groups (Coleoptera, Diptera, Lepidoptera, Hymenoptera, Heteroptera, Neuropterida). In total, we sampled 5716 insects of which 2725 individuals were sampled using the manually operated traps and 2991 by the automated traps. There are no systematic differences between the two trap types (Fig 3). Although comparing only two automated and two manually operated traps do not allow for formal statistical inference, the pilot study confirmed that our approach to automated insect sampling is technically feasible and reliable, resulting in comparable amounts of caught insects. A larger study was conducted with 20 automated insect traps during 104 nights in 2019 in the town of Weiningen (ZH), Switzerland. The traps were mounted directly to the street-light poles at a height of ca. 3 m above ground. Between May 20 and August 31 2019, we caught 49613 insects with an overall mean of about 23 insects per night. If we had relied on sampling with manually operated insect traps during the field assistants' working hours (four nights per week, Monday-Friday), we would have sampled 60 nights in total. However, the automated insect traps allowed us to increase the sampled nights to 104 nights (Monday-Sunday, seven nights; additionally also during public holidays), which represents an increase of 40% in the number of nights sampled. Overall, only one trap repeatedly failed during the trial study and had to be replaced. All traps were restarted weekly to readjust their time measurements. Initial humidity issues in the electronic part of the traps could be solved by sealing the electronic devices with lacquer and drilling ventilation holes into the plastic casing containing the electronic devices (S1 Appendix). After these improvements we did not encounter problems and the traps ran smoothly for more than three months.

## Discussion and conclusion

Insect biodiversity is threatened worldwide and not only insect specialist species with narrow ecological niches are affected, but also generalist species [8, 13, 14]. The environmental factors leading to insect decline are manifold [15] and include loss of habitat caused by urbanization [16, 17], climate change [18], extreme weather conditions (e.g., droughts [19, 20]), or intensive agricultural management, e.g. using pesticides or fertilizers [14, 18, 21]. Detailed knowledge on the individual drivers of insect decline are prerequisite [8, 9, 15] to develop mitigation strategies for a sustainable future. To this end, an efficient, automated insect flight-interception trap may contribute to efficiently monitor and survey insect abundance in changing environments. The automated insect trap presented here allows to sample insects during seven discrete, user-defined time intervals. The time intervals may allow to identify more precisely the effects of individual environmental drivers such as e.g., fertilizer impact, weather conditions, or street lights which likely impact nocturnal insects. Furthermore, diurnal temporal fractioning of insect sampling may allow to temporally better target diverse measures such as agricultural harvesting (e.g. mowing), better focused pesticide treatment, or even adjusted flushing intervals of hydro-dams to avoid emergence times of aquatic insects. While some of such timings may be well known, the pure existence of time-controllable aerial insect traps will enhance research opportunities and to resolve the phenology of focus species much better in terms of time.

We operated the traps during 2018 (71 nights) and 2019 (104 nights) with only an initial failure of one prototype. In the Zürich region, both years where distinctly warmer relative to the long term average of 9.4°C between 1981 and 2010 (2018: +1.7°C; 2019: +1.2°C; https://www.meteoswiss.admin.ch/home/climate/the-climate-of-switzerland/monats-und-jahresrueckblick.html). Nevertheless, the cups to catch insects never dried out. Occasional thunderstorms could cause individual cups to overflow, but the sturdy construction of the rotating trap was never affected. The light-weight PET polytrap devices above the turntable containing the cups would probably be more prone to damage in heavy storms, but we never experienced any problems. Overall, we feel the traps could be used in very different also harsh and remote areas, probably excluding tropical regions with regular heavy rainfall, where this type of device might overflow too regularly. Prerequisite is a fixed position (branch, tree, pole) to carry the construction (trap empty weight 3.6 kg).

Our automated time-interval insect trap addresses several sampling challenges, while ensuring the same trappability as an existing and tested commercial flight-interception trap: (1) the trap can be programmed to sample insects during any time interval of interest, e.g., from sunset to sunrise, or seven 2-hour fractions during daytime, including adjusted sampling-time intervals to changing day-lengths during the sampling period. This enhances sampling accuracy and saves time and personnel costs; (2) the trap connects to smartphones and can be controlled via Bluetooth (S1 Appendix); (3) as all traps start and terminate sampling automatically and simultaneously, the comparability between (distant) experimental sites is ensured; (4) the straightforward mechanical and electronic design of the sampling units are well accessible and easy to handle; (5) the rotating sampling trap could also be mounted to other trapping devices [7].

The traps are relatively expensive at around 700 Euros. However, given that field assistants cost between 2000 and 3000 Euros per month, the automated traps will allow considerable savings in personal resources. In addition, in our sampling setup, the automated traps also sampled during week-ends and public holidays, resulting in at least a 40% larger sample size. The investment in automated traps will therefore pay off in the long term. Finally, the price of the traps is in the same range as other electronic outdoor devices in the data-logger sector.

We believe that in the context of increasing environmental stressors, standardized, user-defined sampling intervals become increasingly important and supplement information obtained from long-term insect monitoring. Our automated insect trap reduces maintenance efforts and allows for a broad range of timely applications to assess environmental impacts on insect diversity in arbitrary temporal fractions.

## Supporting information

S1 Appendix (DOCX)

S2 Appendix (PDF)

S3 Appendix (PDF)

S4 Appendix (HEX)

S5 Appendix (PDF)

S6 Appendix (ZIP)

S7 Appendix (ZIP)

S1 Data (XLSX)
