# Supplementary material for: Automated flight-interception traps for interval sampling of insects
Source: PLoS One. 2020 Jul 10;15(7):e0229476. doi: 10.1371/journal.pone.0229476 (PMC7351151; doi:10.1371/journal.pone.0229476)
Supplement: S1 Appendix — (DOCX) [file pone.0229476.s001.docx]

Appendix A

In the following, we give an exhaustive description of the automated insect traps including all electronic components, their positioning on a printed circuit board, and the layout of the latter. We further describe the setting of the trap manually or through a software on a mobile device. Finally, we illustrate all mechanical parts of the trap, including detailed plans and technical information for CAD software. Professional electronic and mechanic knowledge is required to build the traps. We encourage the readers to contact the authors for support if there are questions.

Electronic Appendix Insect Trap

# Parts description

The electronics of the insect trap include the following parts:

- ultra-low-power Cortex M4 Micro Controller Unit (MCU) with Real-Time Clock (RTC) & crystal oscillator
- low voltage stepper motor driver
- Bluetooth low energy module
- hall switch (detects magnetic field for rotational positioning)
- ultra-low-power DC/DC converter

The DC/DC converter creates and stabilizes the 3.3V supply for all digital devices. The motor is operated with battery power. The ultra-low-power MCU is the heart of the circuit and controls all functions. The MCU runs a finite state machine which selects the distinct actions. The position of the turntable can be detected by the hall switch. It changes its state, when one of the two magnets, attached to the turntable, passes by the hall switch.

Detailed information about electronics parts are given in Appendices B to F.

- **AppendixB** - Electronics scheme.pdf : illustrates the electronic components and circuitry.
- **AppendixC** - Finite state flowchart.pdf : illustrates the software logic of the firmware.
- **AppendixD** - Firmware.hex : contains the firmware in binary format.
- **AppendixE** - Bill of Materials.pdf : lists all parts incl. specifications, manufacturer parts number, etc. as ordered from [https://www.mouser.ch](https://www.mouser.ch/)
- **AppendixF** - Electronics printed circuit board (PCB) layout GERBER.zip : contains the layout of the PCB in Gerber format, an open ASCII vector format for printed circuit board (PCB) designs.

# Controls description

The insect trap can be controlled either by using a software application (APP) on the smartphone or by manually rotating the internal turntable, which is holding the cups, to a specified position. Few settings like the system time and a position reset after a system restart are only available through the APP. To control the insect trap via APP, the mobile phone needs to be connected to the insect trap via Bluetooth.

When the trap is turned on, it moves the turntable according to the preset temporal lookup-table to rotate a cup into position (Pos. 1 to 7) or the pass-through hole (Pos. 8) under the polytrap (Fig. A1). Presently, the lookup-table is fixedly programmed into the firmware. Entries in the lookup-table include the following parameters: day of the year (1 to 366), position (1 to 8, where 8 is the position of the pass-through hole), hour (0 to 23) and minute (0 to 59). The trap stays in the *off* position, when turned off.

# Using the APP to control the insect trap

As long as no Bluetooth device is connecting to the insect trap, the Bluetooth circuitry will switch for 10 seconds in every minute into an ‘advertising mode’. In this mode, the controller is visible for other Bluetooth devices to which a connection can be established. The ‘advertising mode’ is kept short to save energy. The trap can be connected at a distance of up to 25 m, depending on type of phone and unobstructed line of sight. The most stable connections are achieved from a distance of 5-10 m. The transmitting power of the Bluetooth module can be increased if necessary.

To connect to the insect trap circuit, the ‘BGX Commander’ APP from Silicon Laboratories is required. It is freely available on the Apple Store for iOS phones (<https://itunes.apple.com/US/app/id1350920514?mt=8>) or the Google Play Store for Android phones (<https://play.google.com/apps/testing/com.silabs.bgxcommander?pli=1> ).

Silicon Laboratories give a short introduction under ‘<https://docs.silabs.com/gecko-os/1/bgxhost/framework/latest/bgx-commander-overview>‘.

After starting the APP, it is possible to scan for trap devices within Bluetooth reach, which ‘advertise’ themselves (see above), and then select the insect trap to connect to. To connect to a trap, a password is required. Presently this is set fixedly in the firmware to the number ‘726072‘. When the mobile phone is connected to an insect trap, a command window on the APP will open, prepared to accept commands. The entire software control of the insect trap is done by single-line terminal text commands via this window. Sending e.g. the command ‘*help*‘, followed by a return will show all available commands. Table A1 lists all possible commands.

**Table A1**: Listing of Bluetooth commands accepted by the trap and their respective functional response.

| **Command** | **Function executed** |
| --- | --- |
| *help* | shows all available commands |
| *get status* | shows the state of the trap including time, date, battery state, which cups are full and more |
| *set time hhmmss* | set the time to the committed value |
| *set date ddmmyy* | set the date to the committed value |
| *reset pos* | reset the turntable to a known position (required if the turntable has been moved manually) |
| *turn on* | reset the state of the cups (now all empty), reset the position to a defined value and change to on state after disconnect |
| *turn off* | set the trap to off state after disconnect |
| *run motor* | turn the table in counter clockwise direction until the command *stop motor* is transmitted |
| *stop motor* | stops the running motor |
| *selftest* | during a self-test, a position reset is performed and then each position is approached once. IMPORTANT: DO NOT INTERRUPT THIS TEST (CLOSE APP, SEND OTHER COMMANDS, ETC.) |
| *turn ref on* | (only for developer) |
| *turn ref off* | (only for developer) |
| *run steps cw XXXX* | turn the table for XXXX steps in clockwise direction where 1000 steps are a full turn; XXXX is a number between 1 and 9999 (used for developer) |
| *run steps ccw XXXX* | turn the table for XXXX steps in counter clockwise direction where 1000 steps are a full turn; XXXX is a number between 1 and 9999 (used for developer) |

After closing the application, the insect trap changes in the previously chosen state (e.g. *off* or *on*) and rotates the turntable to the appropriate position.

**IMPORTANT**: To switch on the trap it is necessary to set the time and execute a *reset pos* command. This is also required after a battery change or a reset. The status (command *get status*) sent to the insect trap will report, if the *set time* and *reset pos* have been executed once.

# Controlling the insect trap by rotating the turntable to certain positions

The insect trap can be turned **on** and **off** by rotating the trap’s turntable to a specific position. An arrow is marked on the turntable (between Pos. 3 and Pos. 4), indicating the direction. Three labels are attached to the interior of the round sidewall. One label shows the *off* position. The trap will remain in this position if switched off. One of the other two labels reads *turn on*, and the other one reads *turn off*.

When setting up the trap for the first time, these labels will have to be manually attached to the inside wall. Rotate the turntable until the pass-through position is reached (Fig. A1, Pos. 8). Now stick the *turn on* label to the position above cup holder 3, the *turn off* label to the position above cup holder 4 and the *off* label to the position above cup holder 5. Starting or stopping the trap can be triggered manually by setting the arrow either to the *turn on* or the *turn off* position. The turntable will then rotate within one minute to the *off* position (in case *turn off* has been selected) or to one of the cups (in case *turn on* has been selected).


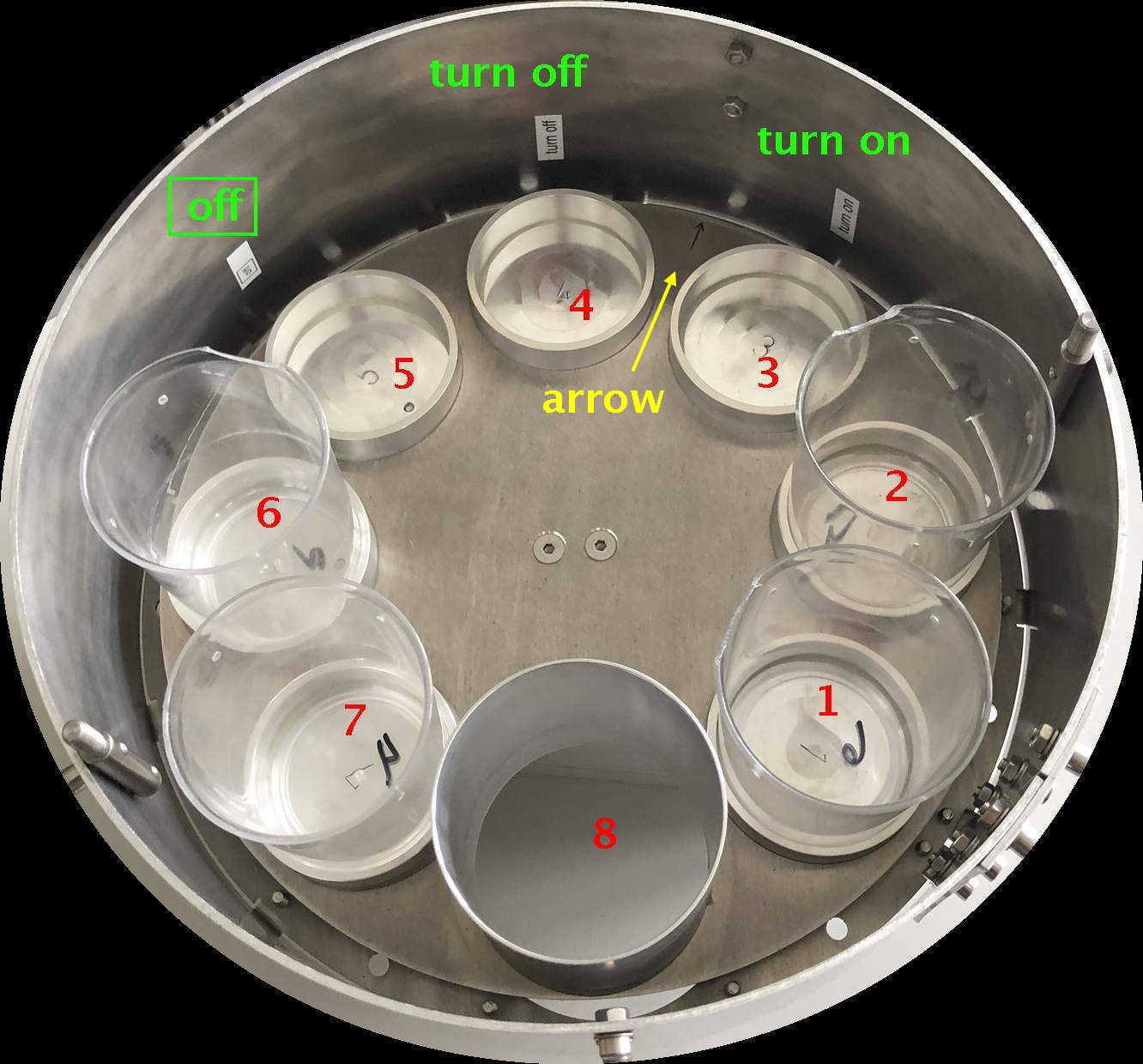


**Figure A1**: Positioning of the labels in relation to the cup locations, when the trap is set to pass-through (Pos. 8).

# Trap full

When the trap has finished a complete sampling round, i.e. all cups are full, it will rotate the turntable to the position with the pass-through opening (i.e., Pos. 8, Fig. A1), also allowing for delayed servicing/emptying of the trap. After emptying the cupts, the trap can be reset either by rotating the turntable to make the arrow point towards the *turn on* label or by sending a ‘*turn on*‘ command with the APP. More information on the trap’s status is transmitted after sending the ‘*get status*’ command on the APP.

# Battery empty

When the battery is almost empty, the trap stays in the pass-through position (Pos. 8, Fig. A1) where no insects are caught. More information on the trap’s status is available after sending the *get status* command on the APP.

# Timetable

Currently, the time at which the turntable moves to another cup or to the pass-through position is fixed in the firmware of the MCU. Times change weekly and are programmed according sunset and sunrise times for Zurich, Switzerland. We encourage readers to contact the authors for respective support in case they want to rebuild/reprogram the construction.

# Protection against rain, humidity and temperatures

Electronics are protected against rain in several steps:

a) all parts are waterproof and made from stainless steel, aluminium, or plastic (POM= Polyoxymethylene)

b) all parts are in a casing below the main trap construction, thus protected from rain,

c) electronic circuits are protected against humidity by spray-on of a protective lacquer.

Ventilation holes are added at the bottom of the casings to allow for air circulation and thus protect the circuitry from condensed water. The casing itself is thus not fully waterproof but allows for air circulation. Building a 100% waterproof casing is possible and would require airtight seals to additionally shield the circuitry from condensation water.

The trap was not explicitly tested for temperatures below 0°C. However, the battery’s operating range is guaranteed between 0°C and 65°C. All electronic components are specified for temperatures between -40°C and 85°C.

# Battery coverage estimation

Batteries to supply electronic circuitries and drive the stepper motor are 1100 mAh Li-Ion. In the presently set configuration (7 nocturnal samplings; 7 diurnal pass-throughs; [=’normal conditions’ = (NC)]) the system needs 26.7 mAh @ 3.7 V per week. Extrapolating over a full half year of operation, the system needs 26.7 mAh x 26 weeks = 694.2 mAh. Thus, for safety reasons, we installed a 1100 mAh battery, allowing for 158% of the minimum required battery capacity. Followingly, under normal conditions [(NC) see above] the battery safely lasts for maximally 41 weeks.

In our setup, two daily operations are envisaged:

a) move turntable to position n (in the evening)

b) move turntable to position ‘pass-through’ (in the morning)

We decisively relate here to our ‘normal conditions’ (NC). If alternate sampling schemes are planned (e.g. several switchings per day), power requirements have to be recalculated accordingly. Power requirements over weeks may vary drastically if multiple daily operations are intended. We encourage readers to contact the authors for respective support in case they want to reprogram the sampling scheme.

# Possible further improvements – not yet implemented

- GPS for automated time calibration
- Custom Application on mobile phones
- Set individual turntable timings in the lookup-table (*)
- Gas gauge sensor to measure used battery capacity
- Brightness sensor for automatic adjustment of the of the lookup-table
- Super cap to bypass power drop in case of a battery change

Neither of these possibilities have been tackled so far. However, we encourage the readers to contact the authors for support, which we will gladly provide, in case they want to rebuild the construction or adapt it (e.g., *).

Mechanical Appendix Insect Trap

To fully explain the building of the mechanical parts of the trap, we have attached ‘**AppendixG** - Mechanical parts.zip’, which contains (a) a read_me.txt file, basically giving the information in Tab. A2, (b), a folder containing the pdfs given in Tab. A2, and (c) a folder ‘step’ containing the file 102472.stp, which contains all mechanical information to be viewed in a CAD software and for laser-processing the mechanical parts described in the pdf's.

**Table A2**: Pdf files contained in the AppendixG (in the pdf folder) and their respective content. Construction material is given in curly brackets, Al = Aluminum, POM = Polyoxymethylene

| **File** | **Content** |
| --- | --- |
| 102472.pdf | The overview document. Starting from here, the working drawing pdf's are referenced and contain, in decreasing numeric order: |
| 102314.pdf | Funnel that externally fits around the PET-insect trap's bottom. Funnel will attach to lid in 102474_1 {Al}. |
| 102368.pdf | Casing for the electronics parts, battery, motor and gears {Plastic}. |
| 102473_11.pdf | Bottom plate of the casing that contains the turntable {Al}. |
| 102473_22.pdf | Side of the casing that contains the turntable. To be attached to 102474_1 {Al}. |
| 102473.pdf | Connecting parts and overview over the casing containing the turntable{Al / 1.4301 / 1.4305}. |
| 102474_1.pdf | Lid of the casing that contains the turntable {Al}. |
| 102474.pdf | Connecting the funnel (102314) to the lid (102474), both {Al}. |
| 102475.pdf | Mechanic parts of the turntable {Al / POM-C / 1.4301 / 1.4305}. |
| 102476.pdf | Bearing for the main axis of the turntable {Al}. |
| 102477.pdf | Mounting of the electric motor with its gear wheel {Al / POM-C} |
| 102478_11.pdf | Turntable {Al}. |
| 102478.pdf | Turntable with positioning of the cups and the pass-through hole {Al}. Note the arrow between Pos. 3 and Pos. 4 on the outer rim! |
