# Supplementary figures and images for: Automated flight-interception traps for interval sampling of insects
Source: PLoS One. 2020 Jul 10;15(7):e0229476. doi: 10.1371/journal.pone.0229476 (PMC7351151; doi:10.1371/journal.pone.0229476)

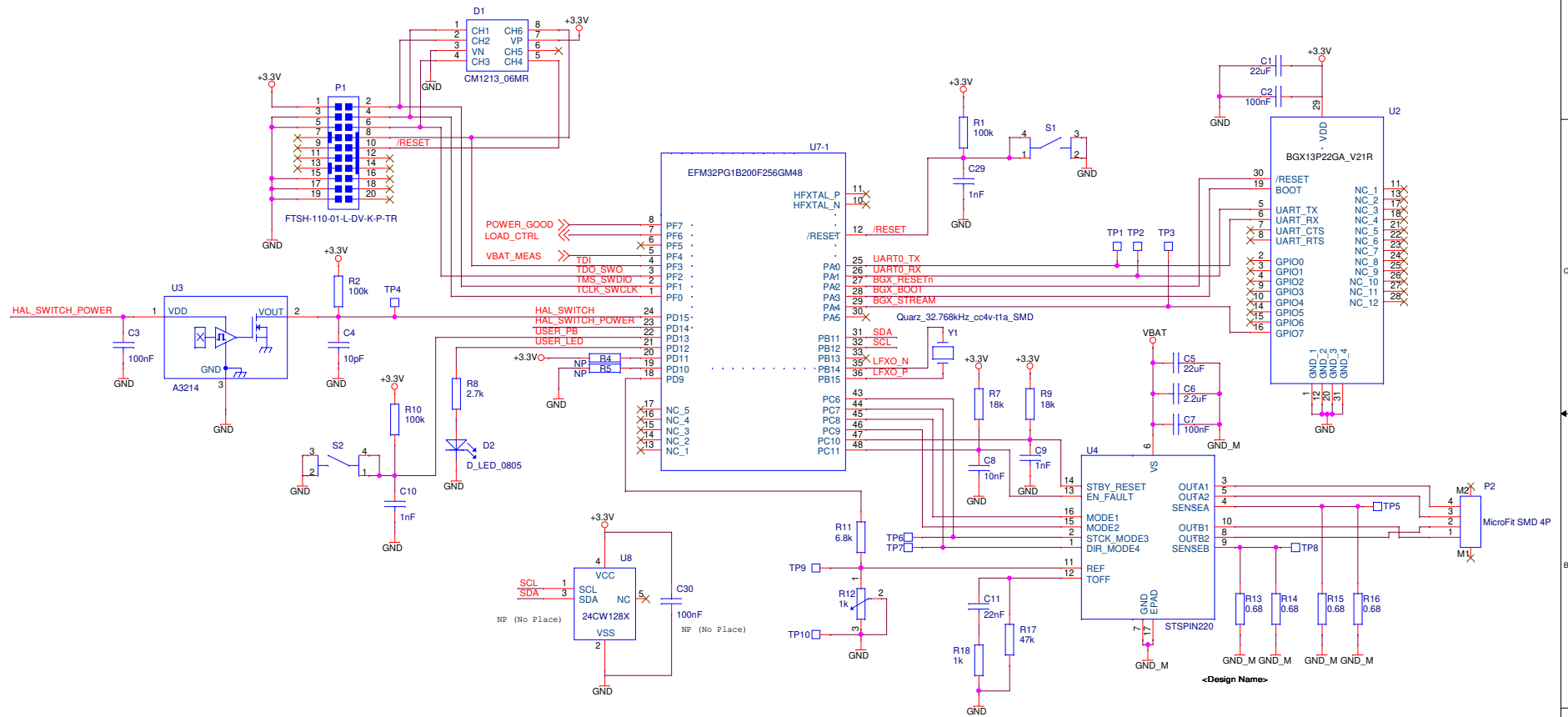

Supplement: S2 Appendix — (PDF) [file pone.0229476.s002.pdf]

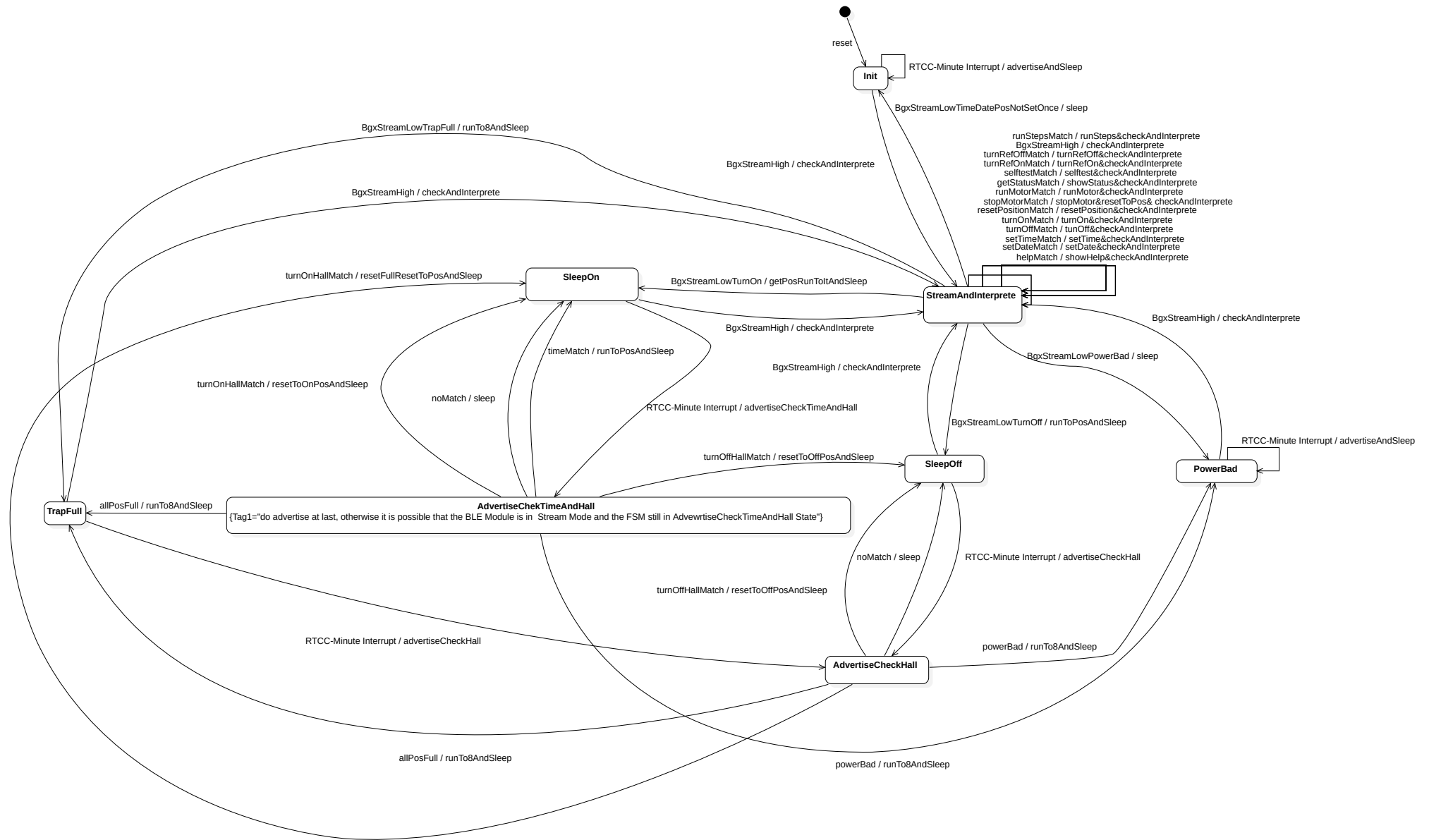

Supplement: S3 Appendix — (PDF) [file pone.0229476.s003.pdf]

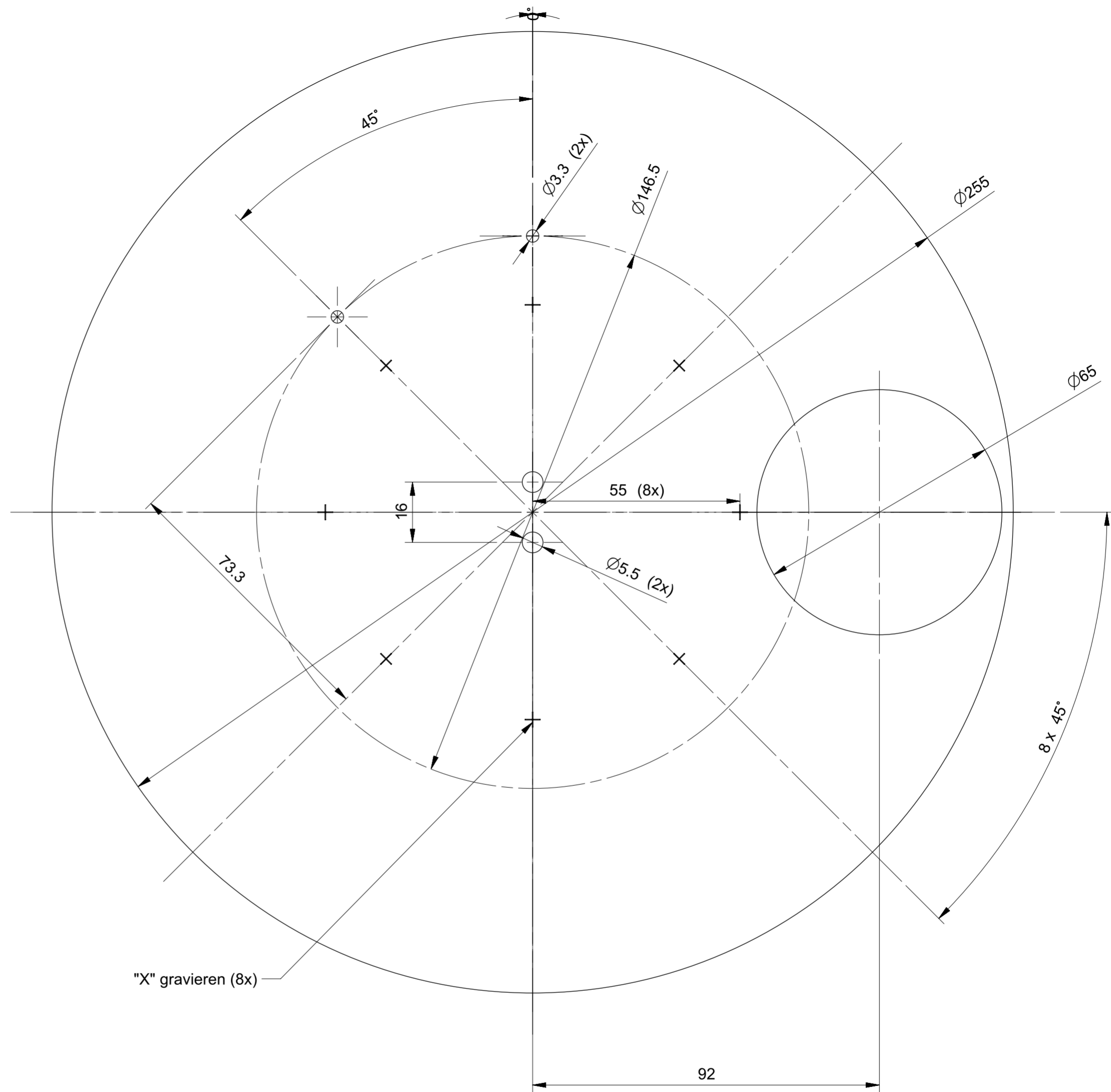[illegible]

Supplement: S7 Appendix — (ZIP) [file pone.0229476.s007.zip › AppendixG - Mechanical parts/pdf/102478_11.pdf]
