# Supplementary material for: Automated flight-interception traps for interval sampling of insects
Source: PLoS One. 2020 Jul 10;15(7):e0229476. doi: 10.1371/journal.pone.0229476 (PMC7351151; doi:10.1371/journal.pone.0229476)
Supplement: S5 Appendix — (PDF) [file pone.0229476.s005.pdf]

Revised: Wednesday, April 29, 2020
Revision: mih
WSL-Institut für Schnee und Lawinenforschung SLF
Bill Of Materials April 29,2020 9:59:16

Item	Quantity	Reference	Part	Manufacturer	Manufacturer Part Number	Place	Supplier Name	Mouser Part Number
1	2	C1,C5	22uF	Taiyo Yuden	EMK316BB7226ML-T	place	Mouser	963-EMK316BB7226ML-T
2	8	C2,C3,C7,C13,C15,C21,C23,C24	100nF	Yageo	CC0603KRX7R9BB104	place	Mouser	603-CC603KRX7R9BB104
3	1	C4	10pF	Murata	GCM1885C2A100FA16D	place	Mouser	81-GCM1885C2A100FA6D
4	1	C6	2.2uF	Yageo	CC1206KKX7R7BB225	place	Mouser	603-CC126KKX7R7BB225
5	3	C8,C18,C19	10nF	Murata	GCM188R72A103KA37J	place	Mouser	81-GCM188R72A103KA7J
6	3	C9,C10,C29	1nF	KEMET	C0603C102J5RACAUTO	place	Mouser	80-C0603C102J5RAUTO
7	1	C11	22nF	Murata	GCM188R72A223KA37J	place	Mouser	81-GCM188R72A223KA7J
8	4	C12,C14,C17,C25	10uF	TDK	C3216X7R1C106K160AC	place	Mouser	810-C3216X7R1C106K
9	1	C16	4.7uF	Taiyo Yuden	EMK212B7475KG-T	place	Mouser	963-EMK212B7475KG-T
10	2	C20,C22	1uF	KEMET	C1206C105K3RAC7210	place	Mouser	80-C1206C105K3R7210
11	1	C26	10uF	TDK	C3216X7R1C106K160AC	no place	Mouser	810-C3216X7R1C106K
12	1	C28	16SVPK100M	Panasonic	16SVPK100M	place	Mouser	667-16SVPK100M
13	1	C30	100nF	Yageo	CC0603KRX7R9BB104	no place	Mouser	603-CC603KRX7R9BB104
14	1	D1	CM1213_06MR	ON Semiconductor	CM1213-06MR	place	Mouser	748-CM1213-06MR
15	1	D2	D_LED_0805	Dialight	598-8150-107F	place	Mouser	645-598-8150-107F
16	1	F1	1206L075THYR	Littelfuse	1206L075THYR	place	Mouser	576-1206L075THYR
17	1	L1	LPS3314 2.2uF	Coilcraft	LPS3314-222MRB	place	Mouser	994-LPS3314-222MRB
18	1	L2	BLM21BD102SN1D	Murata	BLM21BD102SN1D	place	Mouser	81-BLM21B102S
19	1	L3	SRN3015-4R7M	Bourns	SRN3015-4R7M	place	Mouser	652-SRN3015-4R7M
20	1	P1	FTSH-110-01-L-DV-K-P-TR	Samtec	FTSH-110-01-L-DV-K-P-TR	place	Mouser	200-FTSH11001LDVKPTR
21	1	P2	MicroFit SMD 4P	Molex	43650-0424	place	Mouser	538-43650-0424
22	1	P3	MicroFit SMD 2P	Molex	43650-0224	place	Mouser	538-43650-0224
23	1	P4	chassis connection			no place		
24	5	R1,R2,R10,R19,R20	100k	Yageo	RC0603FR-07100KL	place	Mouser	603-RC0603FR-07100KL
25	2	R4,R5	NP			no place		
26	2	R7,R9	18k	Yageo	RC0603FR-0718KL	place	Mouser	603-RC0603FR-0718KL
27	1	R8	2.7k	Yageo	RC0603FR-072K7L	place	Mouser	603-RC0603FR-072K7L
28	1	R11	6.8k	Yageo	RC0603FR-076K8L	place	Mouser	603-RC0603FR-076K8L
29	2	R12,R18	1k	Yageo	RC0603FR-071KL	place	Mouser	603-RC0603FR-071KL
30	4	R13,R14,R15,R16	0.68	Vishay	RCWE2010R680FKEA	place	Mouser	71-RCWE2010R680FKEA
31	1	R17	47k	Yageo	RC0603FR-0747KL	place	Mouser	603-RC0603FR-0747KL
32	1	R21	1R	Yageo	RC0603FR-071RL	place	Mouser	603-RC0603FR-071RL
33	2	R22,R23	R_0603			no place		
34	2	S1,S2	Taster_SMD-Pushbutton	Schurter	1301.9314	place	Mouser	693-1301.9314
35	6	TP1,TP2,TP11,TP12,TP13,TP14	TP_1			no place		
36	9	TP3,TP4,TP5,TP6,TP7,TP8,TP9,TP10,TP15	TP_SMD			no place		
37	1	U2	Value	Silicon Labs	BGX13P22GA-V21R	place	Mouser	634-BGX13P22GA-V21R
38	1	U3	A3214	Allegro Microsystems	A3214ELHLT-T	place	Farnell	
39	1	U4	STSPIN220	STMicroelectronics	STSPIN220	place	Mouser	511-STSPIN220
40	1	U5	MCP1810	Microchip	MCP1810T-33I/TT	no place	Mouser	579-MCP1810T33ITT
41	1	U6	Value	Texas Instruments	TPS62740DSSR	place	Mouser	595-TPS62740DSSR
42	1	U7	EFM32PG1B200F256GM48	Silicon Laboratories	EFM32PG1B200F256GM48-C0R	place	Mouser	634-PG1B200256GM48CR
43	1	U8	24CW128X	Microchip	24CW1280T-I/OT	no place	Mouser	579-24CW1280T-I/OT
44	1	Y1	Quarz_32.768kHz_cc4v-t1a_SMD	ECS	ECS-.327-12.5-39-C-TR	place	Mouser	520-.327-12.5-39-C-T
