# Supplementary material for: Automated flight-interception traps for interval sampling of insects
Source: PLoS One. 2020 Jul 10;15(7):e0229476. doi: 10.1371/journal.pone.0229476 (PMC7351151; doi:10.1371/journal.pone.0229476)
Supplement: S7 Appendix — (ZIP) [file pone.0229476.s007.zip › AppendixG - Mechanical parts/pdf/102368.pdf]

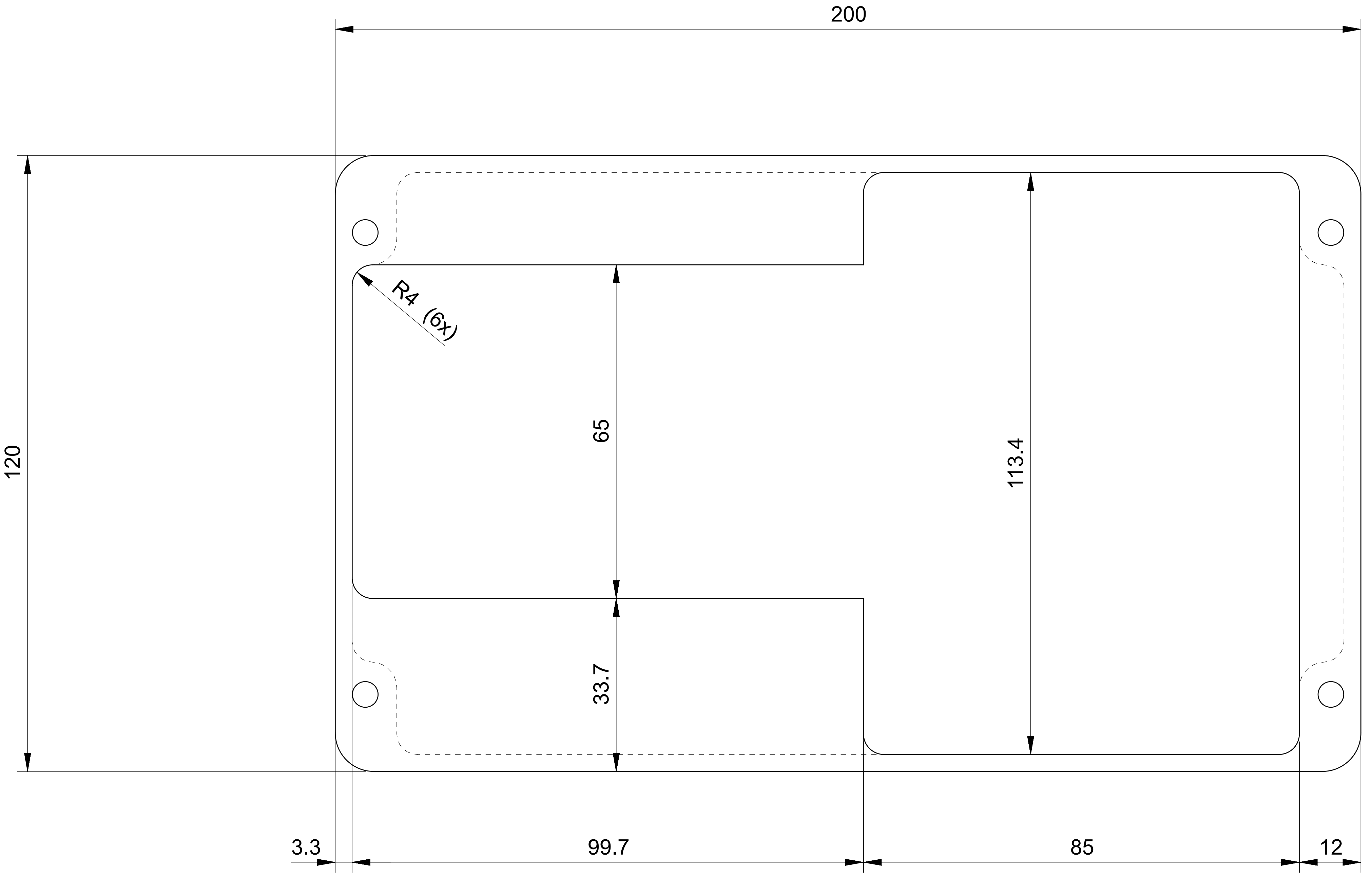

Gehaeuse:
Distrelec Art. Nr. 300-64-392

Achtung Gehaeuse konisch!!!!!!!!

Index	Datum	Name	Änderungen

Werkstoff KST		Ersatz für
Gewicht		Ersetzt durch

Benennung	Massstab		Datum	Name
Kunststoffgehaeuse	1:1	Gezeichnet	05.02.2018	Collet
Insektenfalle		Geprüft		
		Freigeg,		

	Format	Zeichnungs-Nr.	Blatt
WSL-Institut für Schnee- und Lawinenforschung SLF	A3	102368	1/1
