# Supplementary material for: Automated flight-interception traps for interval sampling of insects
Source: PLoS One. 2020 Jul 10;15(7):e0229476. doi: 10.1371/journal.pone.0229476 (PMC7351151; doi:10.1371/journal.pone.0229476)
Supplement: S7 Appendix — (ZIP) [file pone.0229476.s007.zip › AppendixG - Mechanical parts/pdf/102478.pdf]

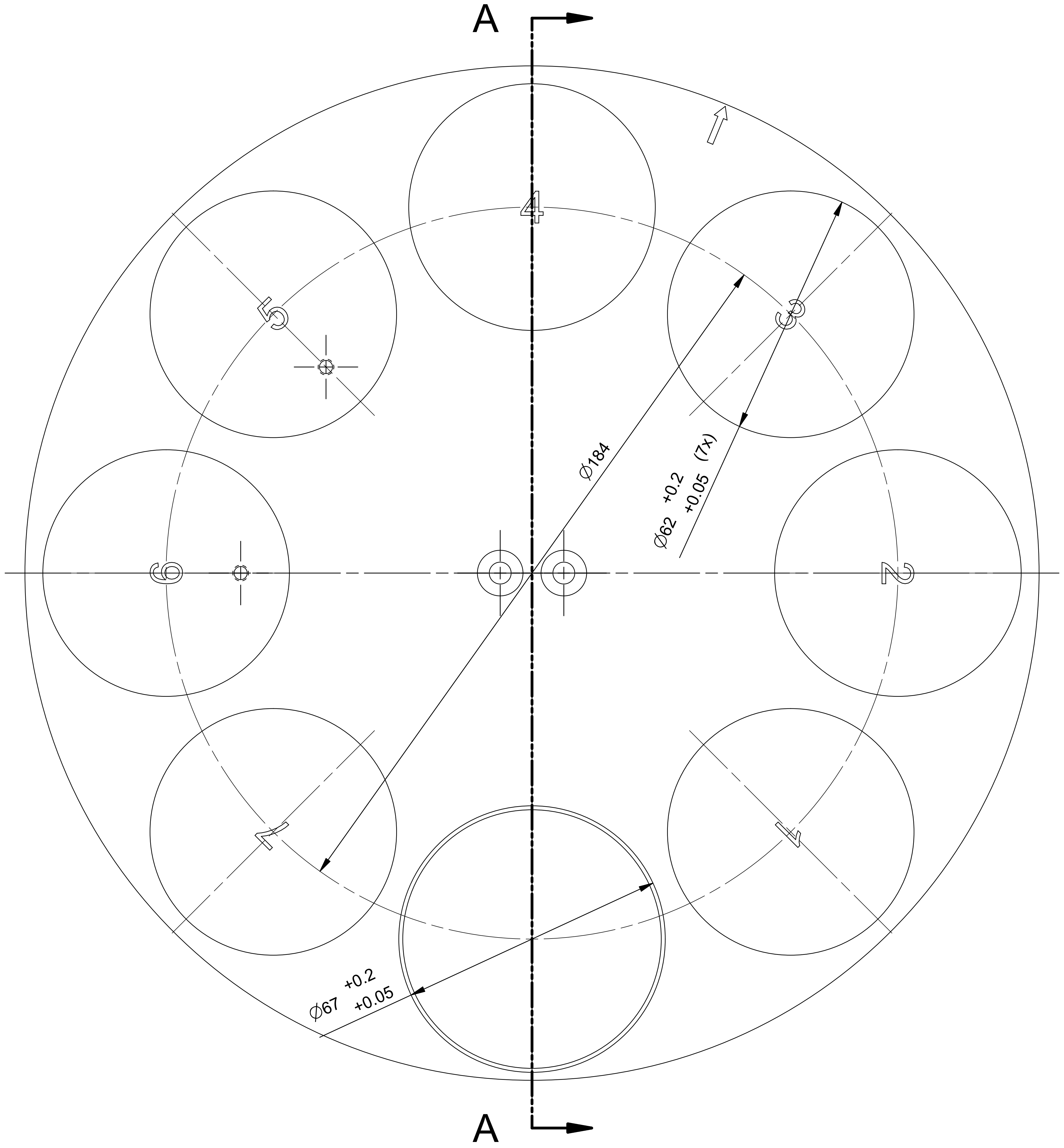

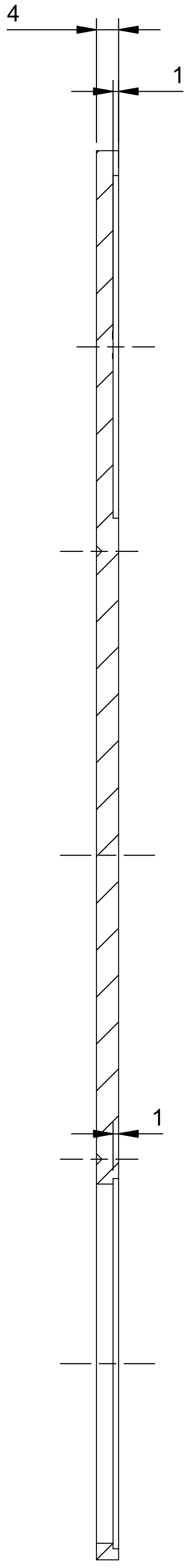

SECTION A-A

Zeichung für Laserfirma 102748_11

Index	Datum	Name	Änderungen		
Werkstoff Alu					Ersatz für
Gewicht					Ersetzt durch
Benennung	Massstab		Datum	Name	
Revolverboden V2	1:1	Gezeichnet	01.03.2019	Collet	
Landschaftsoekologie Insektenfalle		Geprüft			
		Freigeg,			
WSL-Institut für Schnee- und Lawinenforschung SLF	Format A2	Zeichnungs-Nr. 102478			Blatt 1/1
