# Supplementary material for: Automated flight-interception traps for interval sampling of insects
Source: PLoS One. 2020 Jul 10;15(7):e0229476. doi: 10.1371/journal.pone.0229476 (PMC7351151; doi:10.1371/journal.pone.0229476)
Supplement: S7 Appendix — (ZIP) [file pone.0229476.s007.zip › AppendixG - Mechanical parts/pdf/102473.pdf]

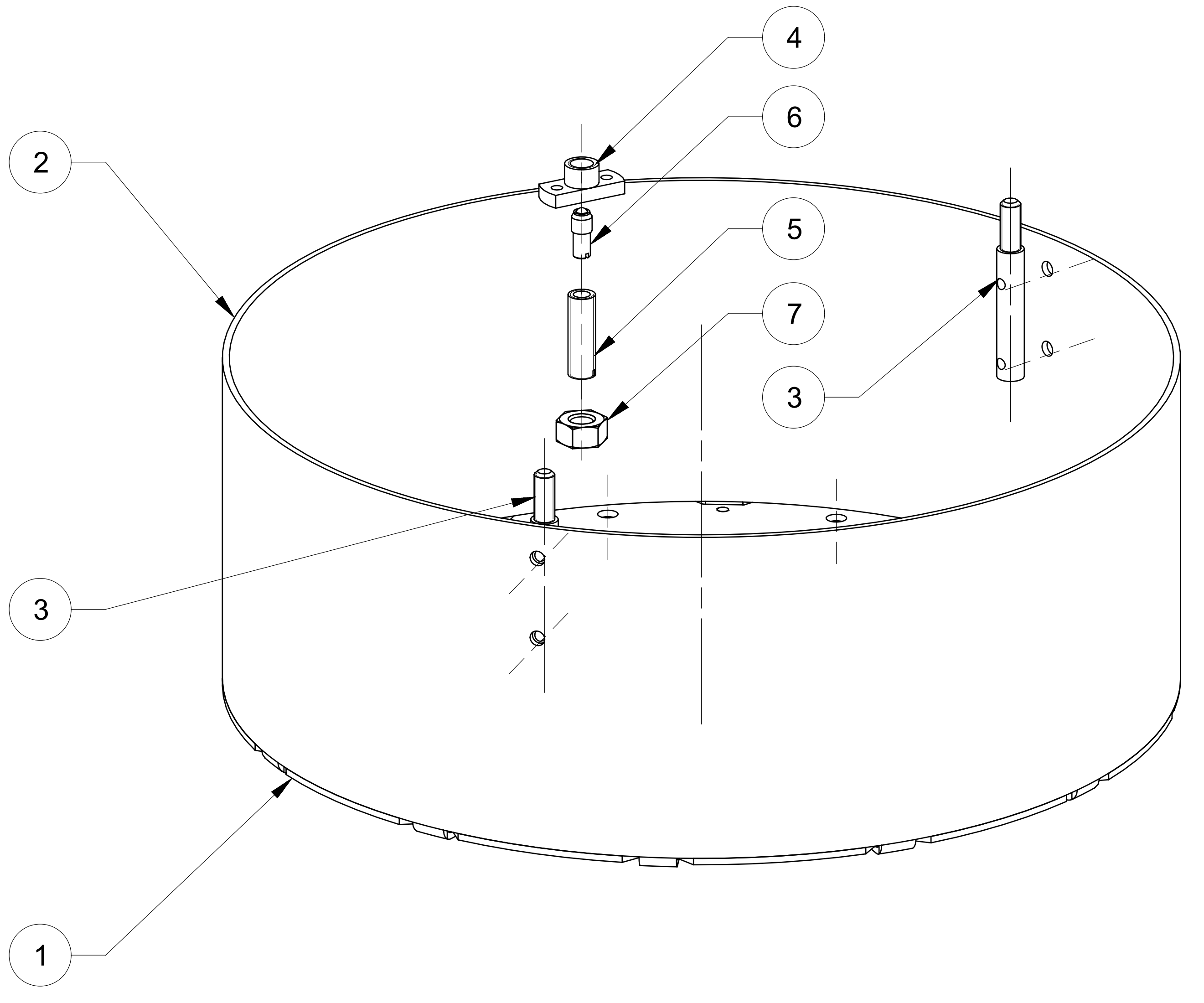

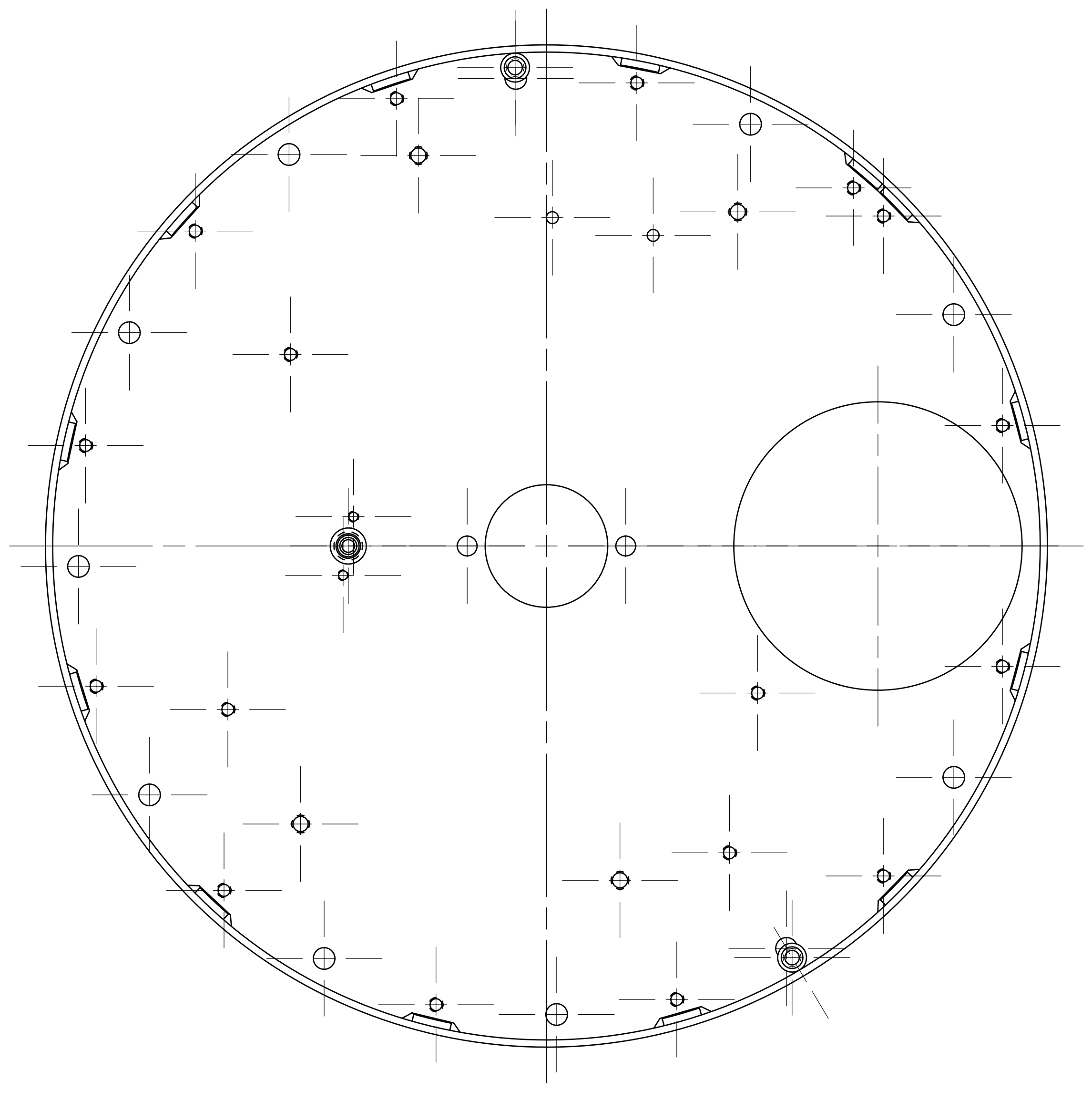

Pos. 3 (1:1) 1.4305 / 1.4301

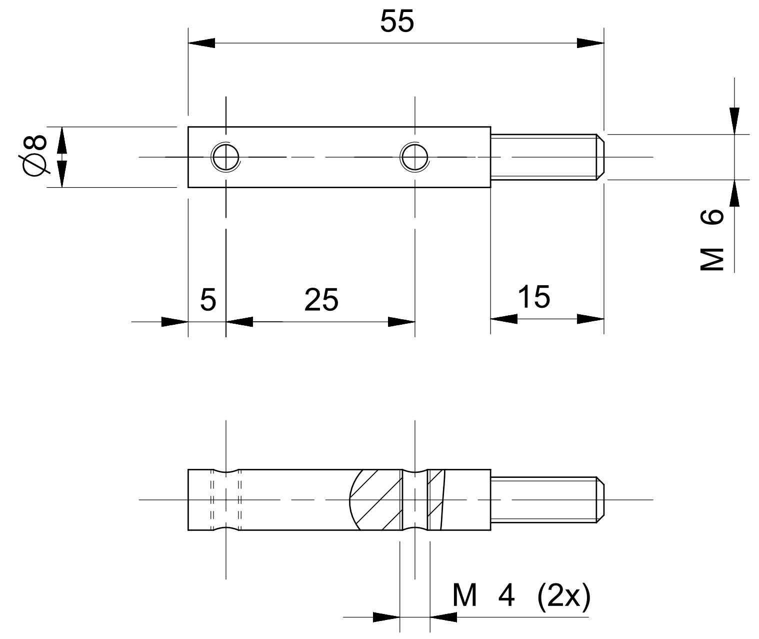

Pos. 4 (1:1) 1.4305 / 1.4301

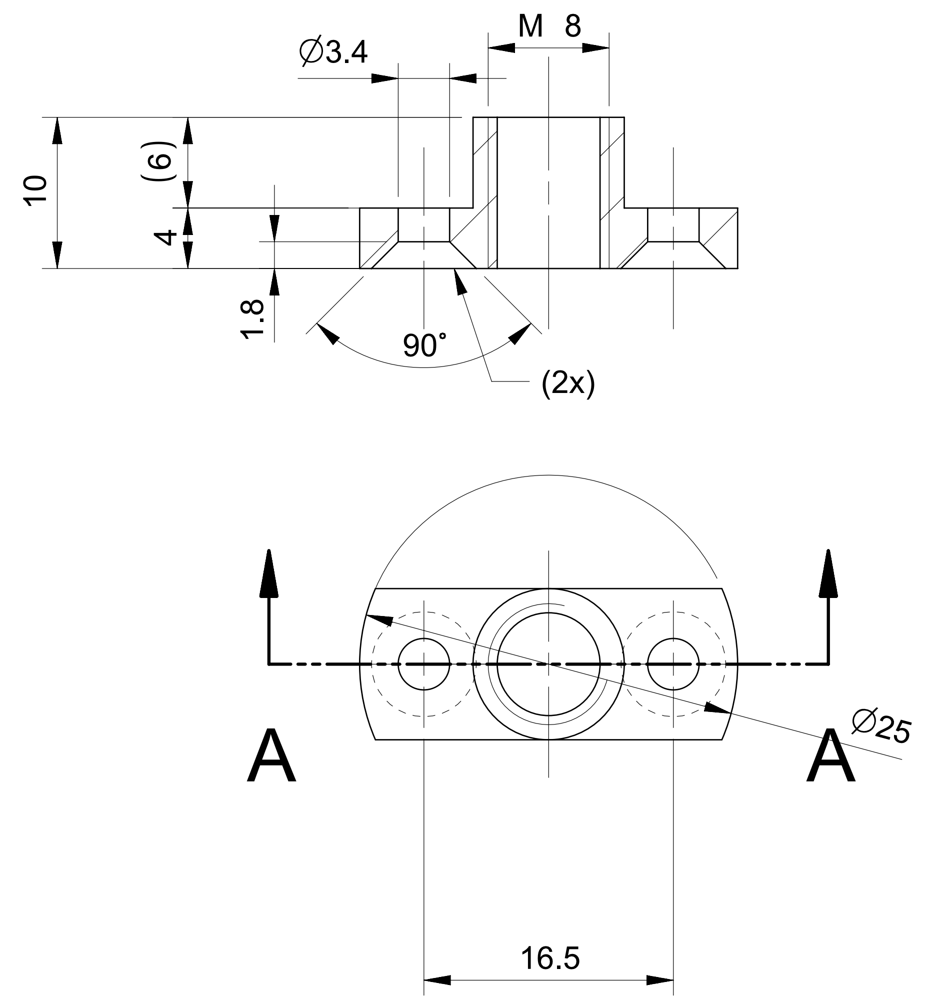

Pos. 5 (2:1) 1.4305 / 1.4301

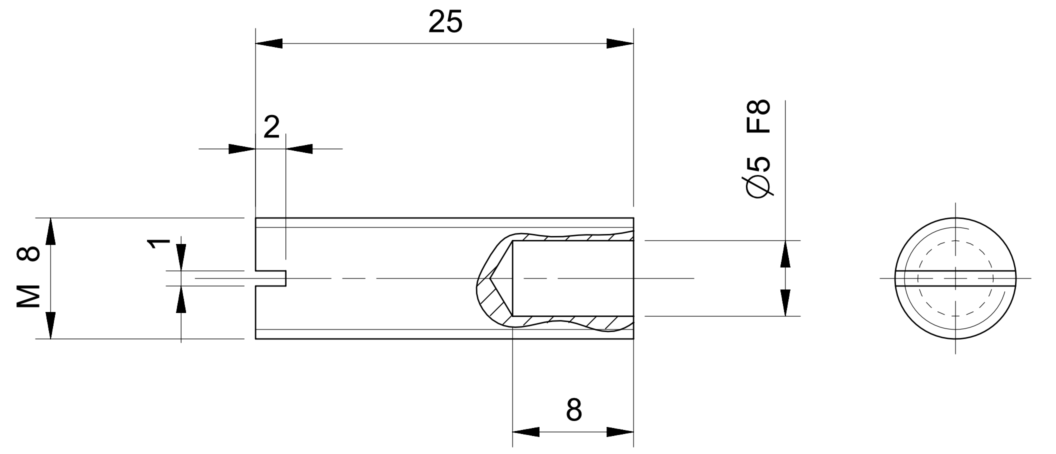

Pos. 6 (2:1)

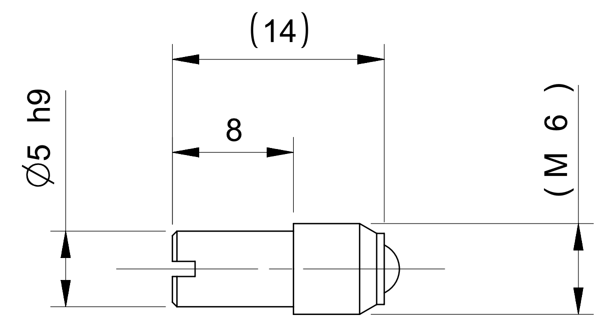

500707
Federndes Druckstück M6 POM-Kugel (bearbeitet); Norelem; 03004-06

Pos.1: - Zeichnung für Laserfirma 102743_11

Pos. 2: - Zeichung für Laserfirma 102473_22
- Achtung beim Biegen. Unbedingt Bigerichtung beachten

Pos Nr	Anzahl	BENENNUNG	Teilenummer
7	1		DIN934-M8X125
6	1		500707_PRT
5	1		102473_5
4	1		102473_4
3	2		102473_3
2	1	Seitenwand	102473_2
1	1	Grundplatte	102473_1

Index	Datum	Name	Änderungen

Werkstoff Alu / 1.4301 / 1.4305		Ersatz für
Gewicht		Ersatzt durch

Benennung	Massstab		Datum	Name
Gehaeuse V2	1:2	Gezeichnet	17.01.2019	Collet
Landschaftsoekologie Insektenfalle		Geprüft		
		Freigeg.		

	Format	Zeichnungs-Nr.	Blatt
WSL-Institut für Schnee- und Lawinenforschung SLF	A2	102473	1/1
