# Supplementary material for: Automated flight-interception traps for interval sampling of insects
Source: PLoS One. 2020 Jul 10;15(7):e0229476. doi: 10.1371/journal.pone.0229476 (PMC7351151; doi:10.1371/journal.pone.0229476)
Supplement: S7 Appendix — (ZIP) [file pone.0229476.s007.zip › AppendixG - Mechanical parts/pdf/102472.pdf]

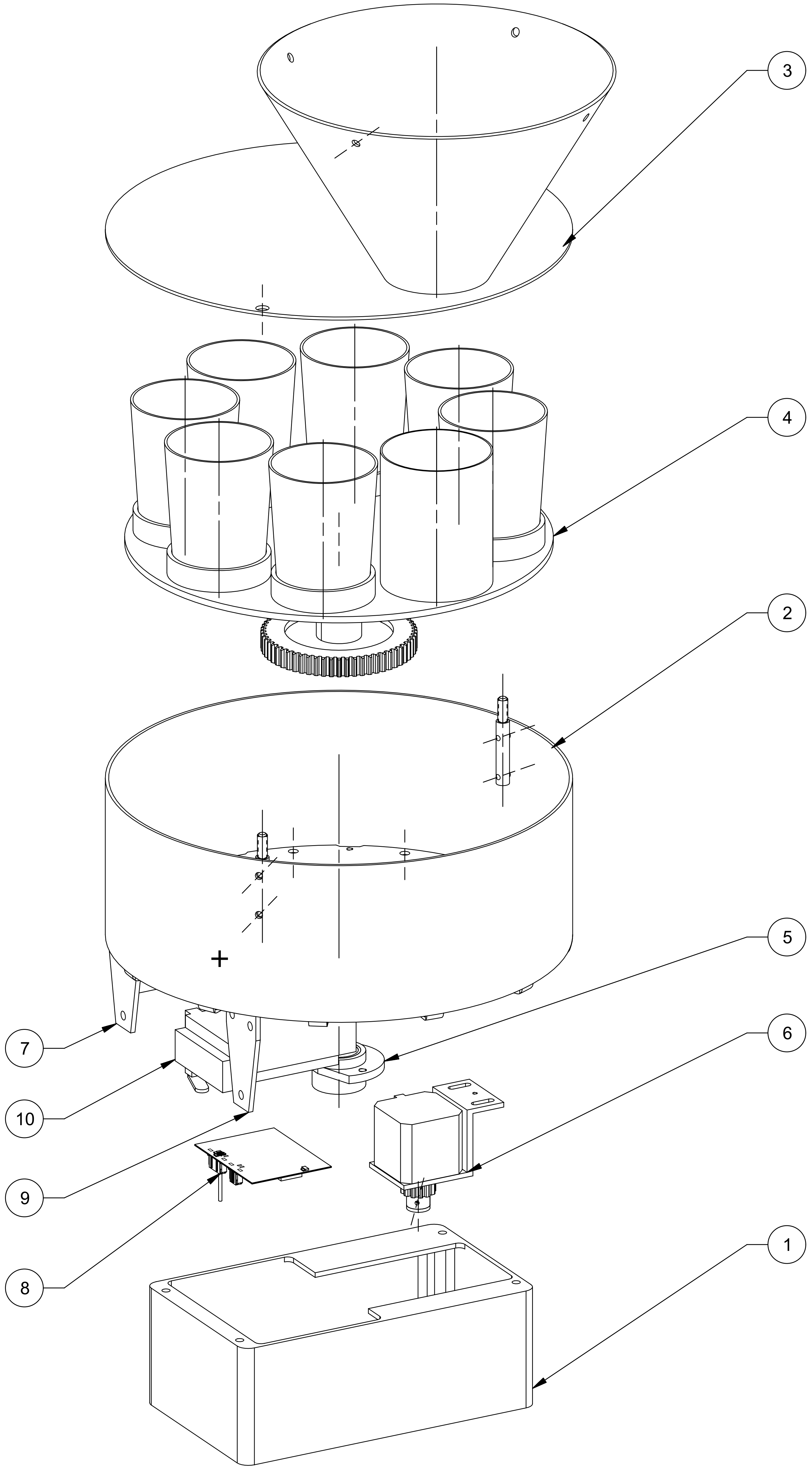

Akku:
Swaytronic LiPo 1S 3.7V 1100mAh 35C/70C JST
Hersteller Art. Nr. 7640159360933
Hersteller: Swaytronic
Lieferant: Conrad Electronic
Bestellnummer Conrad Electronic: 2514522

Buchse Schrittmotor:
Typ: 43645-0400
Hersteller: Molex
Lieferant: Mouser
Bestellnummer Mouser: 538-43645-0400
Kontakte:
Typ: 43030-0008
Hersteller: Molex
Lieferant: Mouser
Bestellnummer Mouser: 538-43030-0008

Buchse Akku:
Typ: 43645-0200
Hersteller: Molex
Lieferant: Mouser
Bestellnummer Mouser: 538-43645-0200
Kontakte:
Typ: 43030-0008
Hersteller: Molex
Lieferant: Mouser
Bestellnummer Mouser: 538-43030-0008

500690
Konsolen HEBGO FL 15; Weber.ch; Artikelnummer 73.5703.0150

500691
Konsolen HEBGO FL 80; Weber; Artikelnummer73.5703.0080

Pos. 8 Insektenfallen PCB

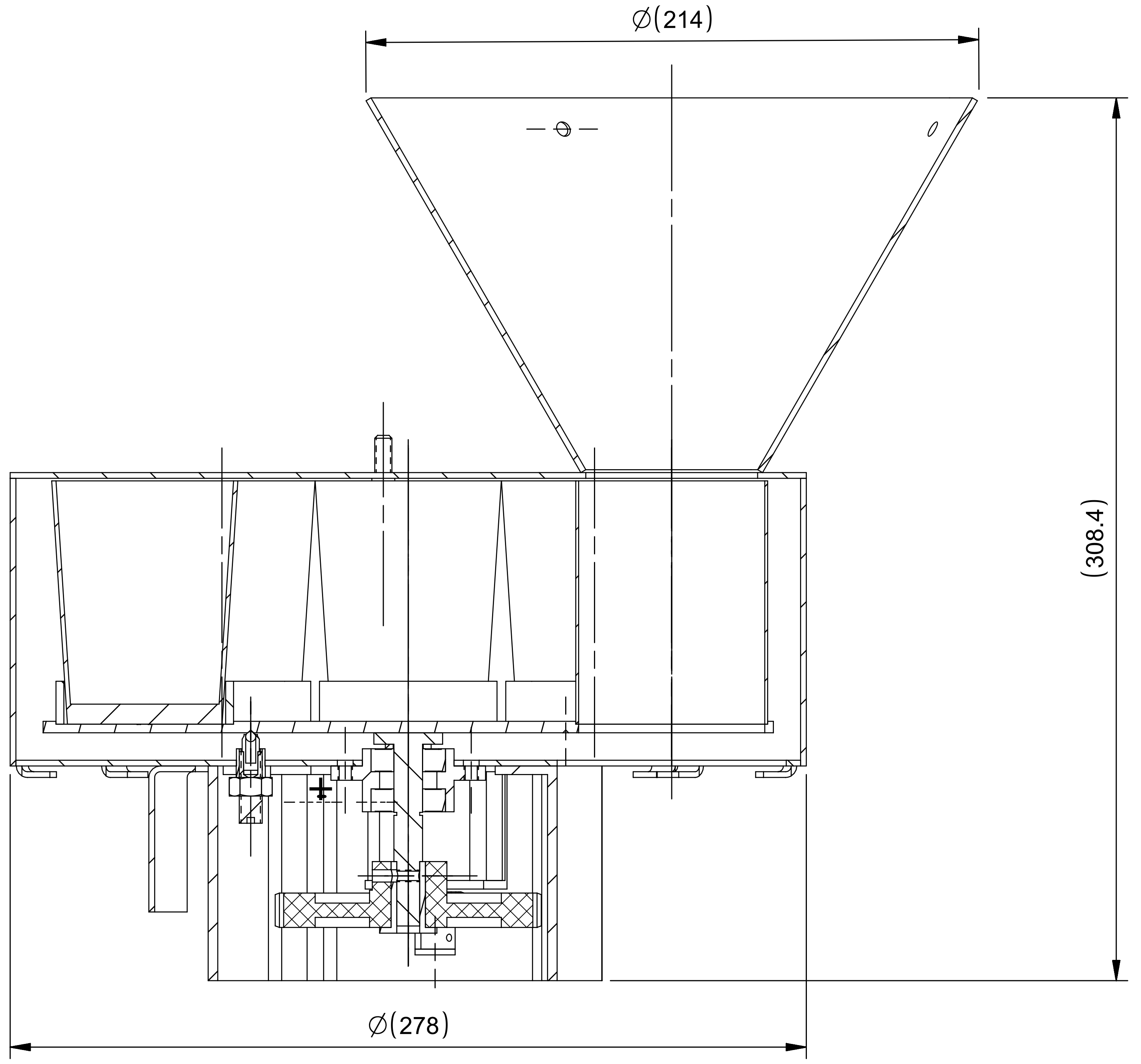

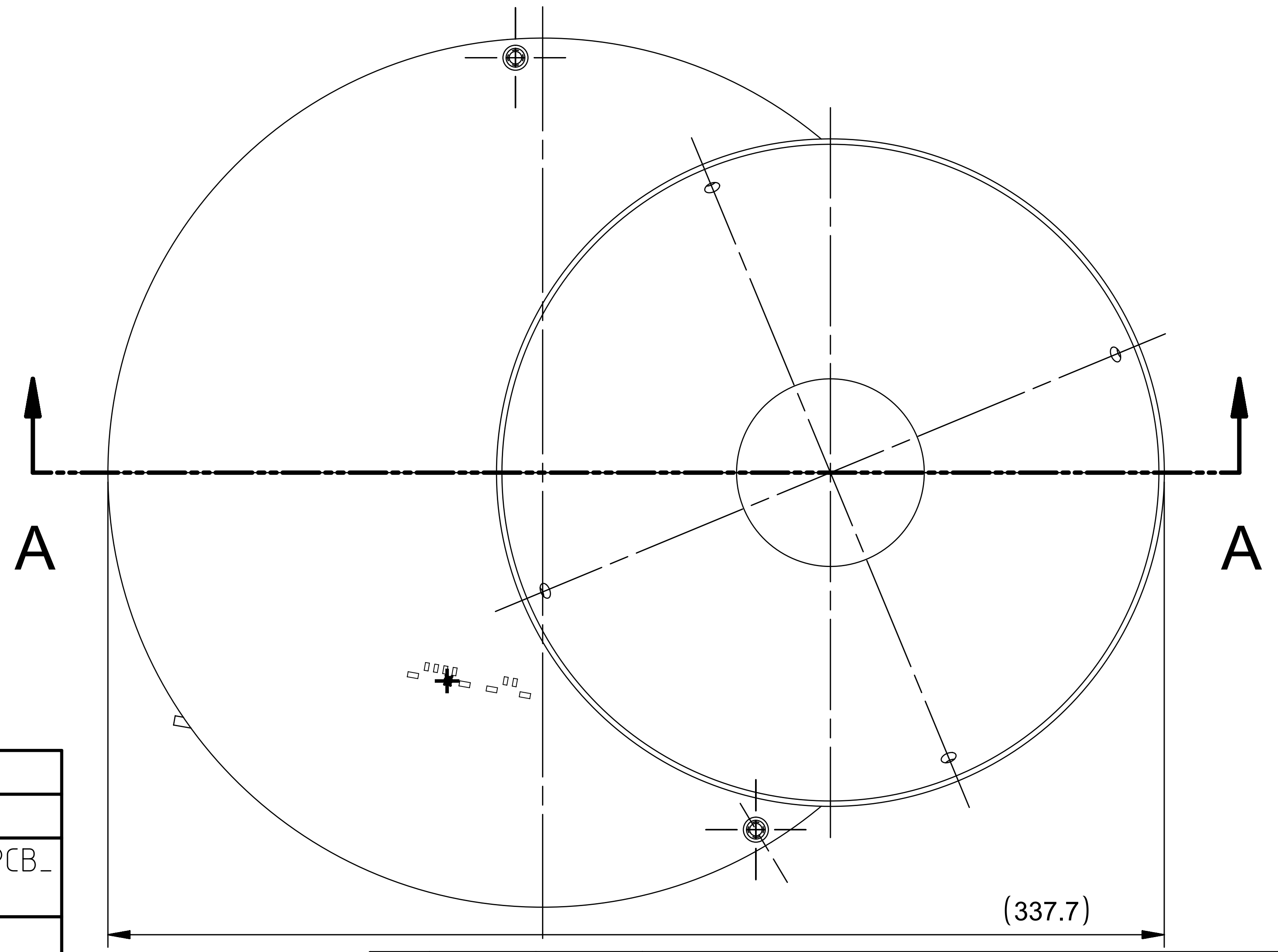

Pos. Nr.	Anzahl	BENENNUNG	Teilenummer
10	1		102539_ASM
9	1		500691_PRT
8	1		INSEKTENFALLE2_PCB_ WO_HOLES_STP
7	1		500690_PRT
6	1	Motoreinheit	102477_ASM
5	1	Lagerung	102476_ASM
4	1	Revolver	102475_ASM
3	1	Deckel	102474_ASM
2	1		102473_ASM
1	1		102368_PRT

Index	Datum	Name	Änderungen

Werkstoff
Gewicht
Ersatz für
Ersatzt durch

Benennung
Insektenfalle V2 kpl
Landschaftsoekologie Insektenfalle

Massstab		Datum	Name
1:2	Gezeichnet	04.02.2019	Collet
	Geprüft		
	Freigeg,		

WSL-Institut für Schnee- und Lawinenforschung SLF

Format: A2
Zeichnungs-Nr.: 102472
Blatt: 1/1
