# Supplementary material for: Automated flight-interception traps for interval sampling of insects
Source: PLoS One. 2020 Jul 10;15(7):e0229476. doi: 10.1371/journal.pone.0229476 (PMC7351151; doi:10.1371/journal.pone.0229476)
Supplement: S7 Appendix — (ZIP) [file pone.0229476.s007.zip › AppendixG - Mechanical parts/pdf/102314.pdf]

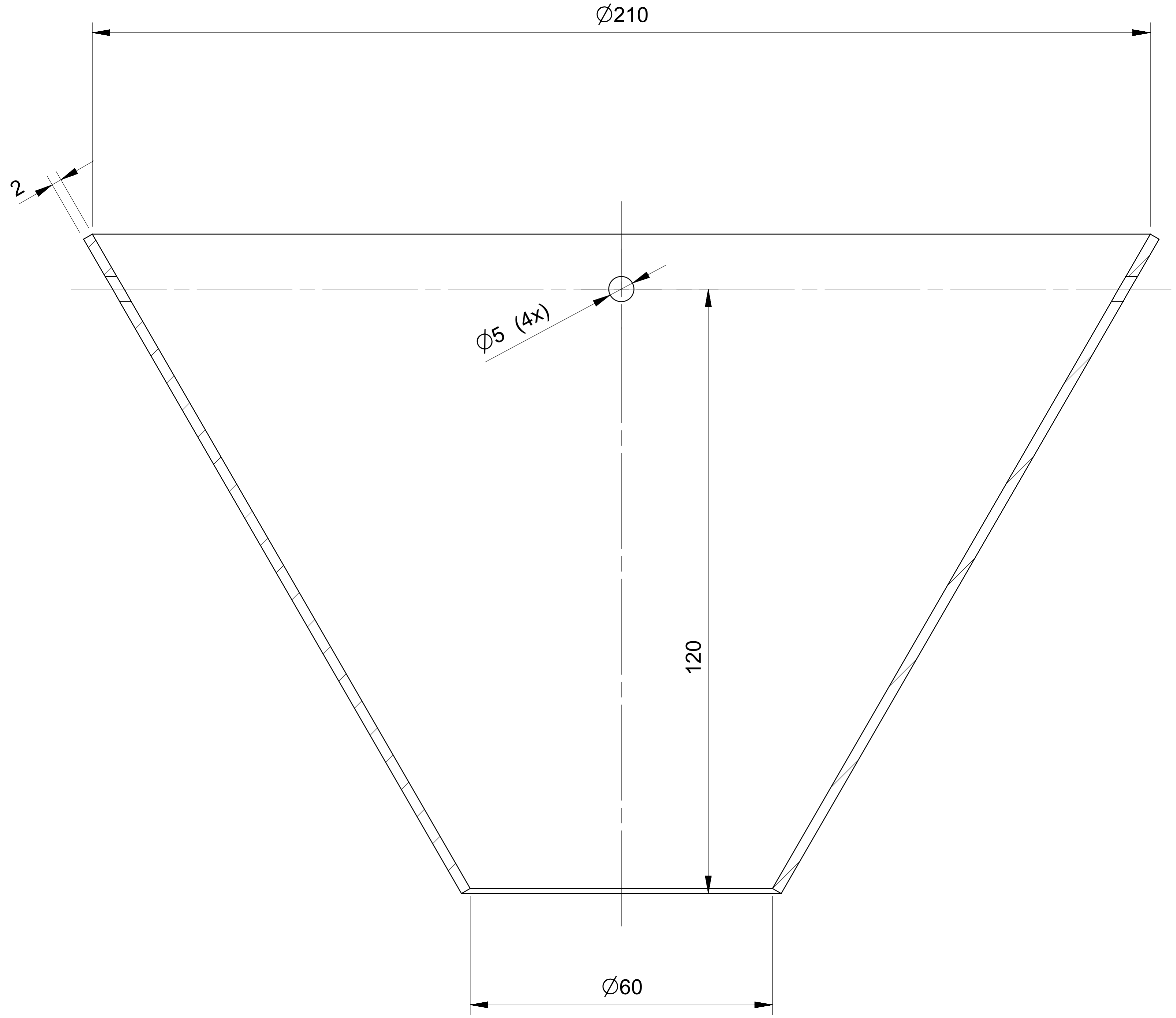

(1:2)

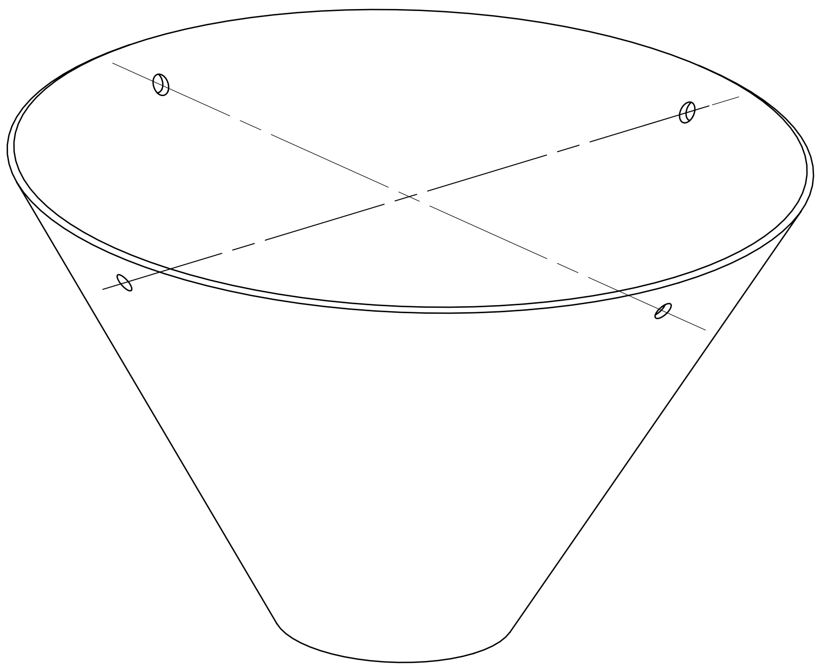

Allgemeintolleranzen ISO 2768-m

Index	Datum	Name	Änderungen

Werkstoff Aluminium		Ersatz für
Gewicht		Ersetzt durch

Benennung	Massstab		Datum	Name
Trichter	1:1	Gezeichnet	13.12.2017	Collet
Insektenfalle		Geprüft		
		Freigeg,		

	Format	Zeichnungs-Nr.	Blatt
WSL-Institut für Schnee- und Lawinenforschung SLF	A3	102314	1/1
