# Supplementary material for: Automated flight-interception traps for interval sampling of insects
Source: PLoS One. 2020 Jul 10;15(7):e0229476. doi: 10.1371/journal.pone.0229476 (PMC7351151; doi:10.1371/journal.pone.0229476)
Supplement: S7 Appendix — (ZIP) [file pone.0229476.s007.zip › AppendixG - Mechanical parts/pdf/102475.pdf]

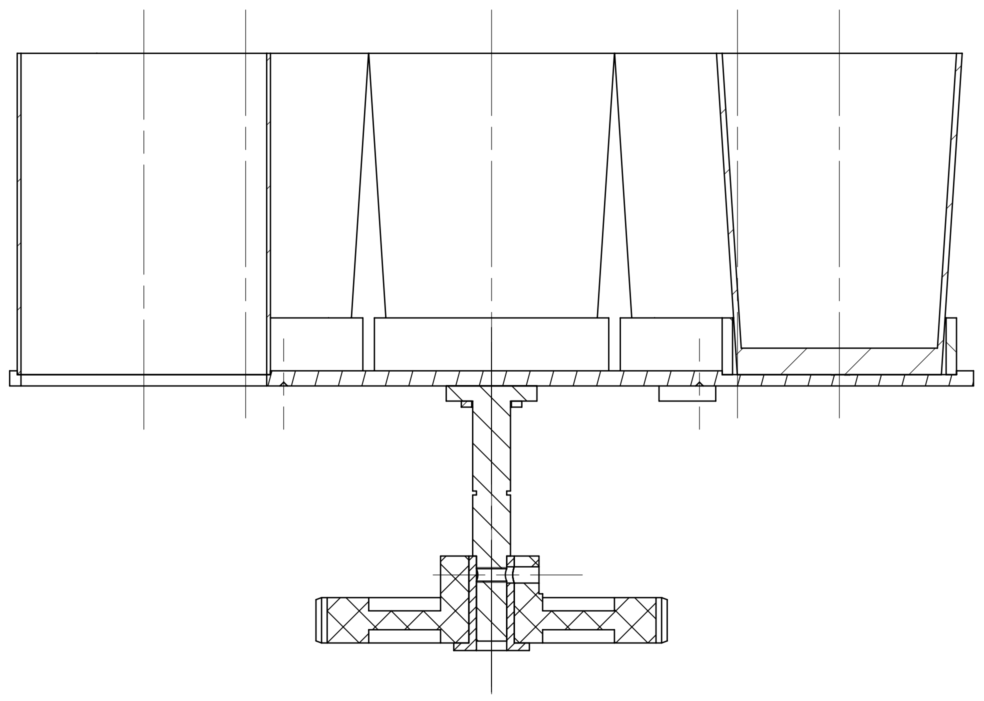

SECTION A-A

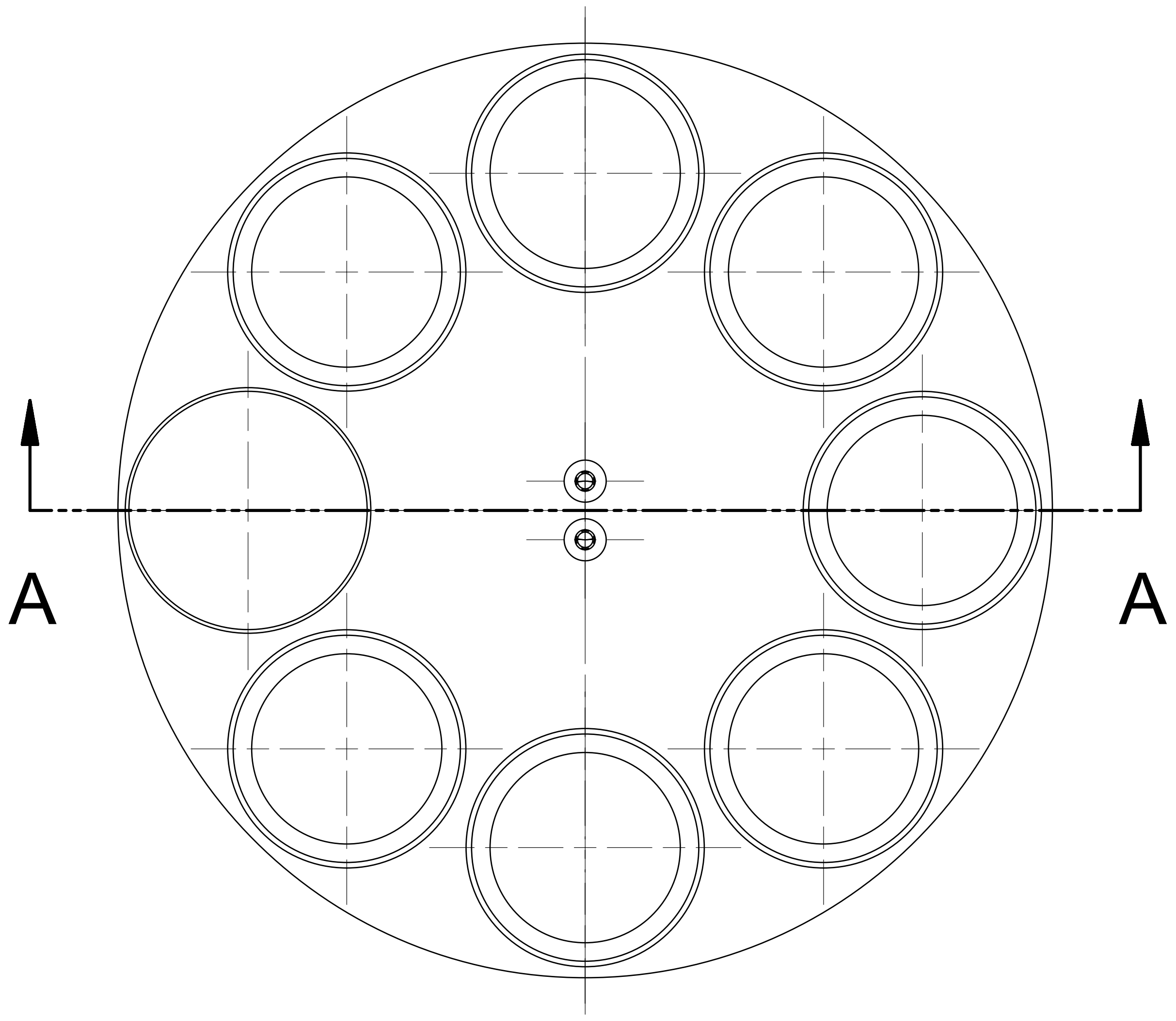

Pos. 1 Alu

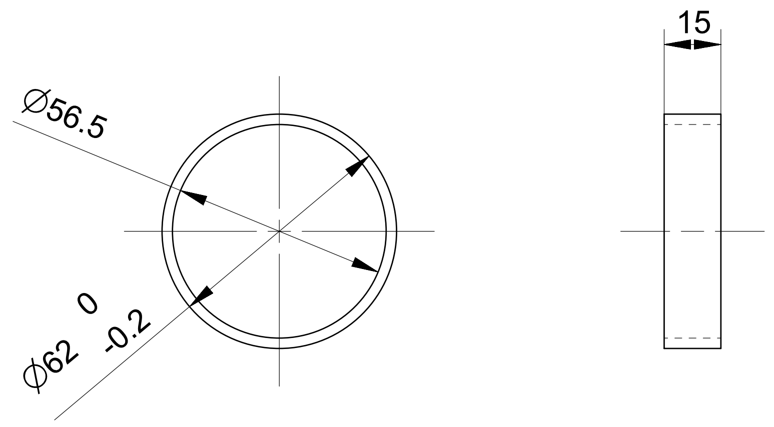

500682
Stirnzahnrad Polyacetal m1,5 60Z D93mm ;
Mädler; Artikelnummer 28506000

500681
Dose PS 0,2 lt; Semadeni; 1925

500724
Scheibenmagnet mit Bohrung und Senkung; D15 h4;
Supermagnete.ch; Artikelnummer CS-S-15-4-N

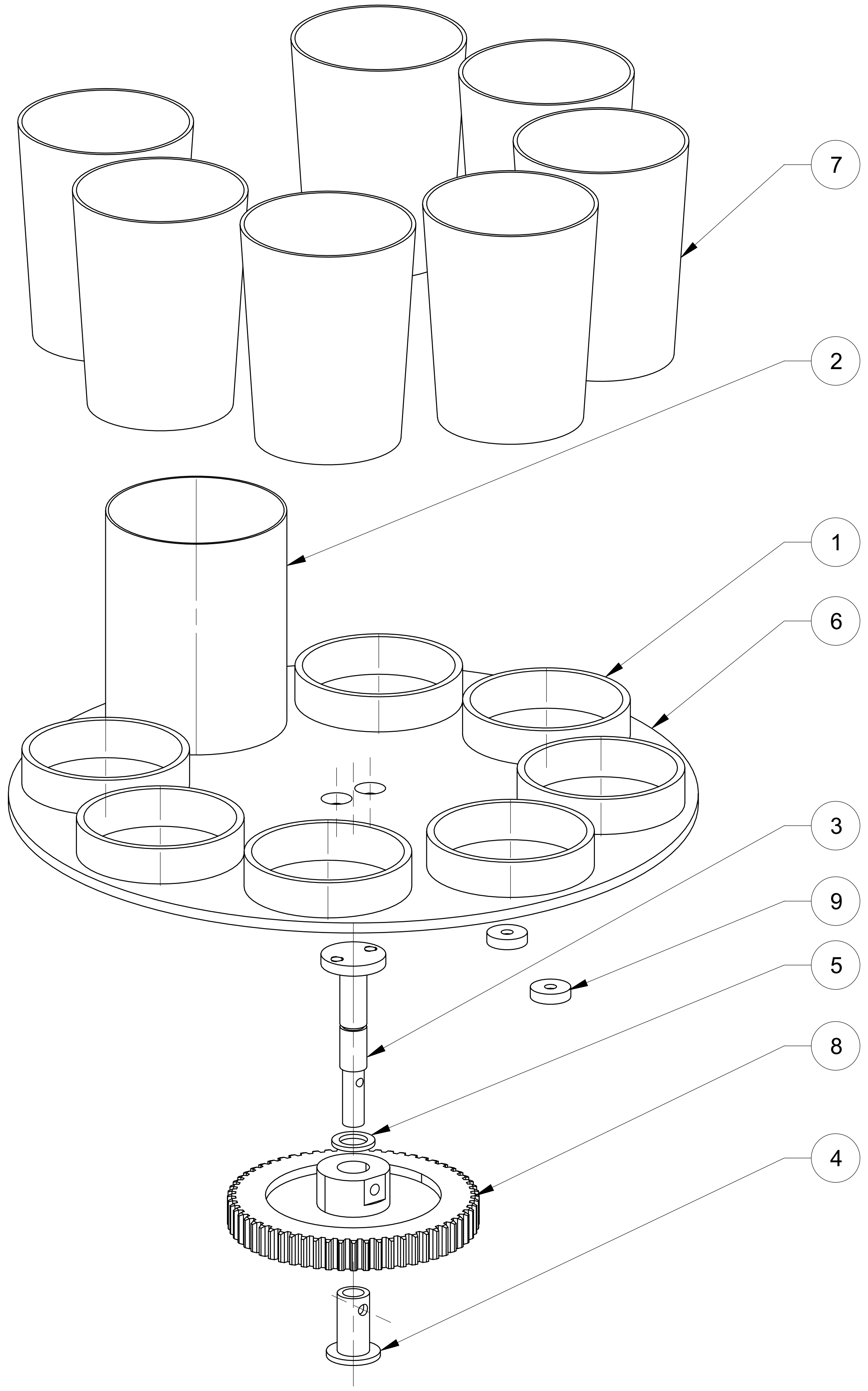

Pos. 2 Alu

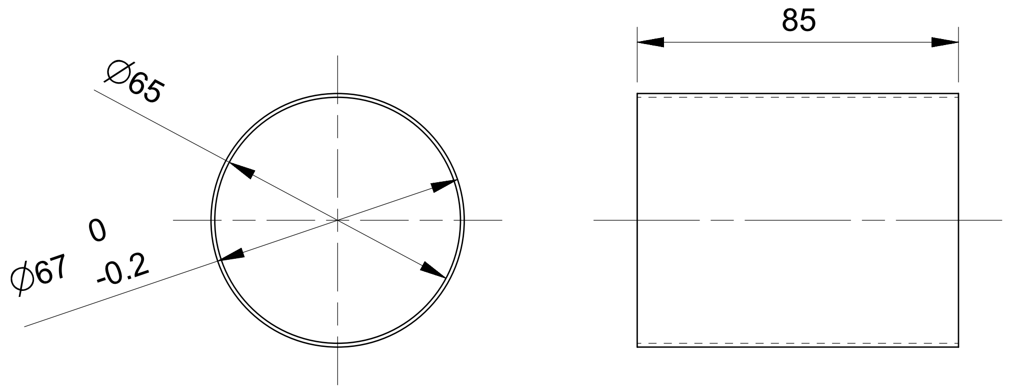

Pos. 3 (1:1) 1.4301 / 1.4305

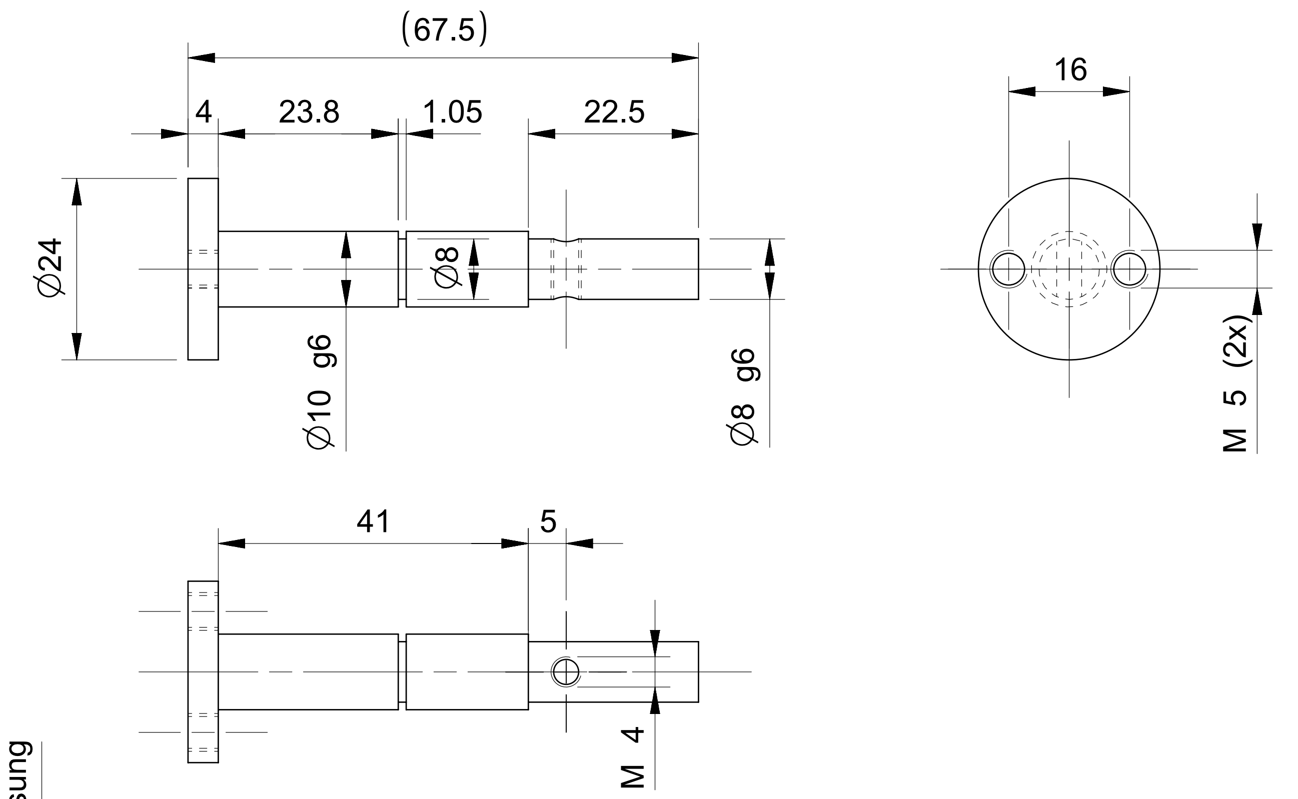

Pos. 8 (1:1) POM-C

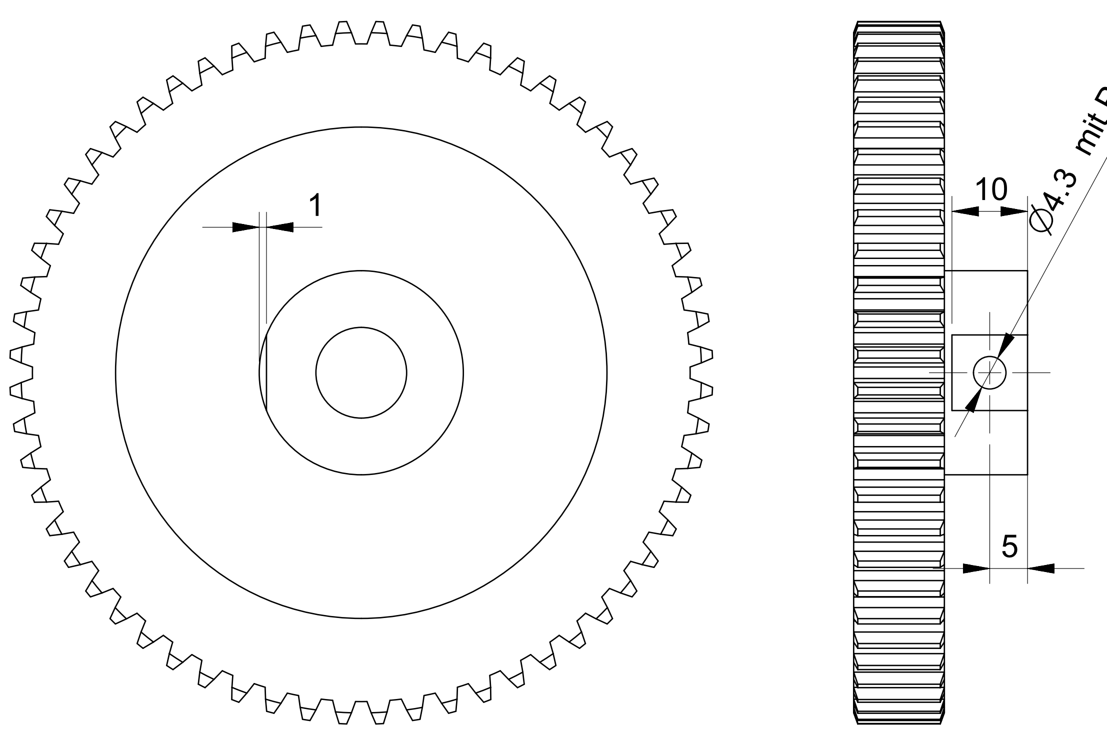

Pos. 4 (1:1) Alu

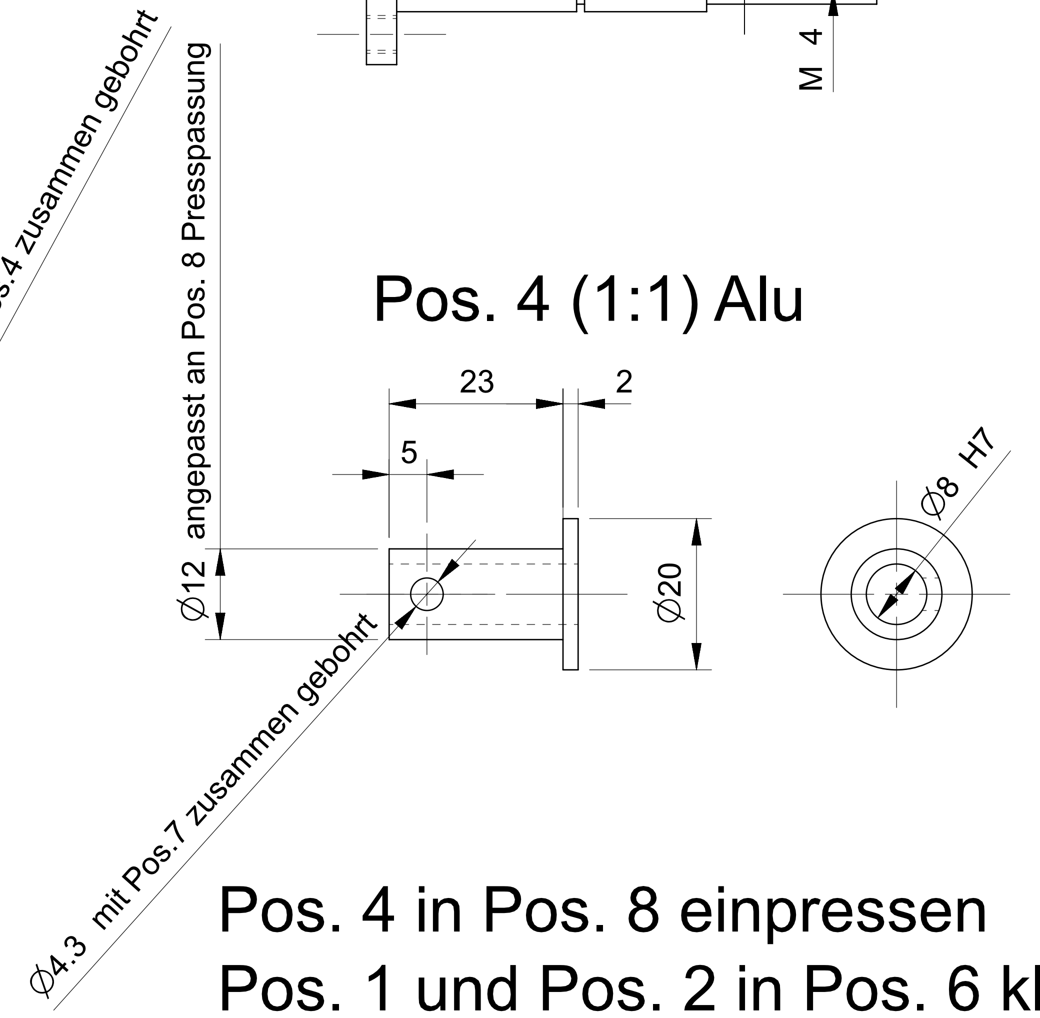

Pos. 5 (A2)

M8 U-Scheibe aufbohren

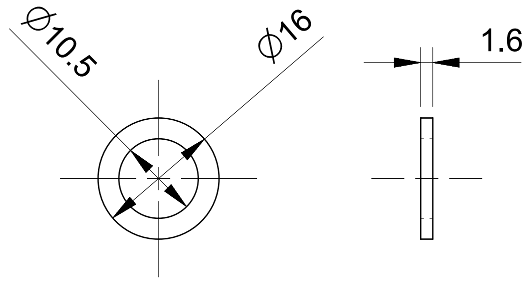

Pos. 4 in Pos. 8 einpressen
Pos. 1 und Pos. 2 in Pos. 6 kleben

Pos Nr	Anzahl	BENENNUNG	Teilenummer
9	2	Scheibenmagnet, D15 h4	500724_PRT
8	1	Stirnzahnrad Polyacetal m1,5 60Z D93mm	500682_PRT
7	7	Dose PS 0,2 lt	500681_PRT
6	1	Revolverboden	102478_PRT
5	1	Distanzscheibe	102475_6
4	1	Huelse	102475_4
3	1	Welle	102475_3
2	1	Durchgang	102475_2
1	7	Becherhalterung	102475_1

Index	Datum	Name	Änderungen

Werkstoff Alu / Pom-C / 1.4301 / 1.4305			Ersatz für		
Gewicht			Ersetzt durch		
Benennung	Massstab		Datum	Name	
RevolverV2	1:2	Gezeichnet	04.02.2019	Collet	
Landschaftsoekologie Insektenfalle		Geprüft			
		Freigeg.			
WSL-Institut für Schnee- und Lawinenforschung SLF	Format A2	Zeichnungs-Nr. 102475			Blatt 1/1
