# Supplementary material for: Automated flight-interception traps for interval sampling of insects
Source: PLoS One. 2020 Jul 10;15(7):e0229476. doi: 10.1371/journal.pone.0229476 (PMC7351151; doi:10.1371/journal.pone.0229476)
Supplement: S7 Appendix — (ZIP) [file pone.0229476.s007.zip › AppendixG - Mechanical parts/pdf/102474.pdf]

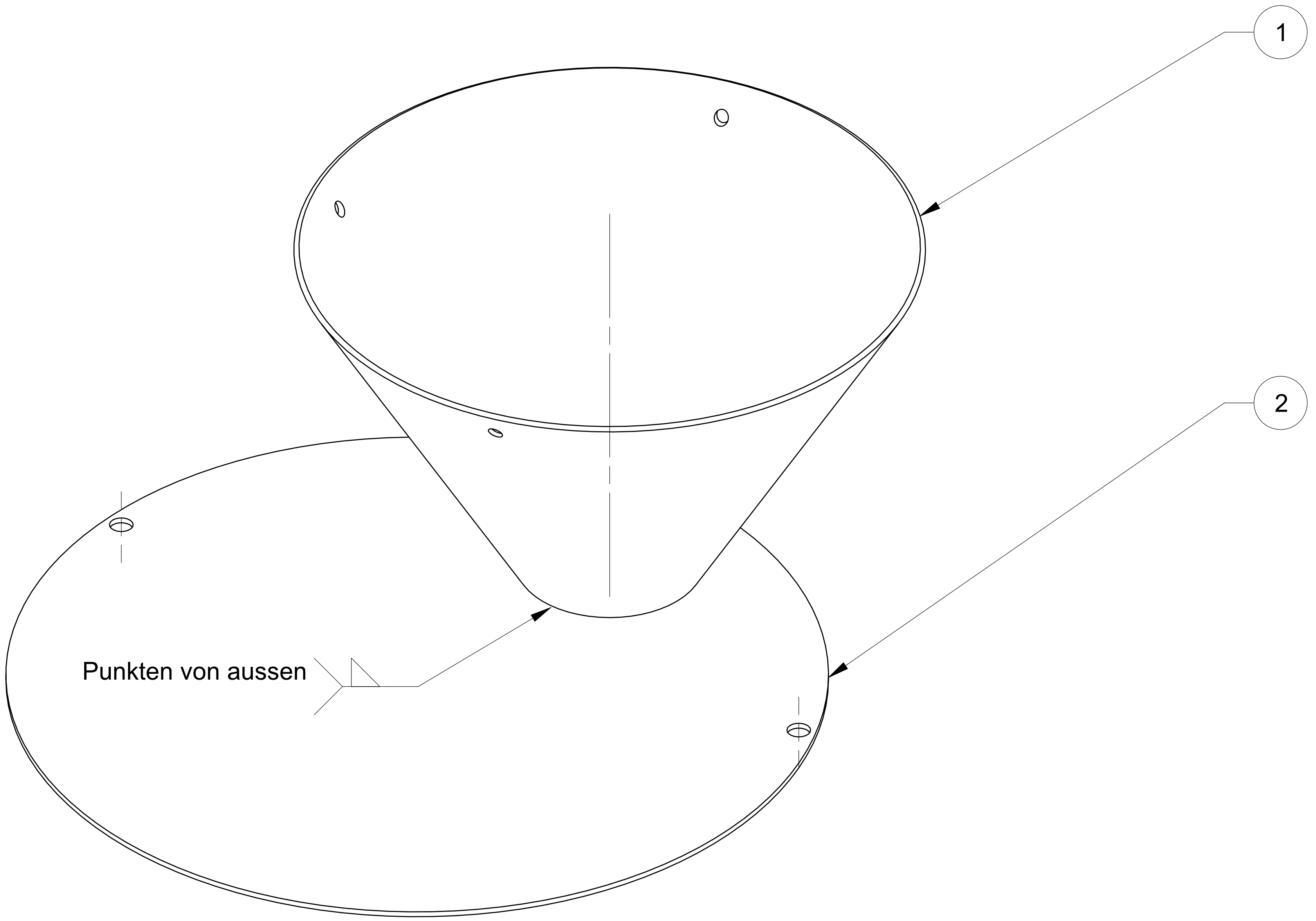

Pos. Nr.	Anzahl	BENENNUNG	Teilenummer
2	1		102474_1
1	1		102314_PRT

Index	Datum	Name	Änderungen

Werkstoff Alu	Ersatz für
Gewicht	Ersetzt durch

Benennung	Massstab		Datum	Name
Deckel V2<br>Landschaftsoekologie Insektenfalle	1:2	Gezeichnet	04.02.2019	Collet
		Geprüft		
		Freigeg,		

	Format	Zeichnungs-Nr.	Blatt
WSL-Institut für Schnee- und Lawinenforschung SLF	A3	102474	1/1
