# Supplementary material for: Automated flight-interception traps for interval sampling of insects
Source: PLoS One. 2020 Jul 10;15(7):e0229476. doi: 10.1371/journal.pone.0229476 (PMC7351151; doi:10.1371/journal.pone.0229476)
Supplement: S7 Appendix — (ZIP) [file pone.0229476.s007.zip › AppendixG - Mechanical parts/pdf/102476.pdf]

500683
Rillenkugellager 6000-SKF; Mädler; Artikelnummer 6000-SKF

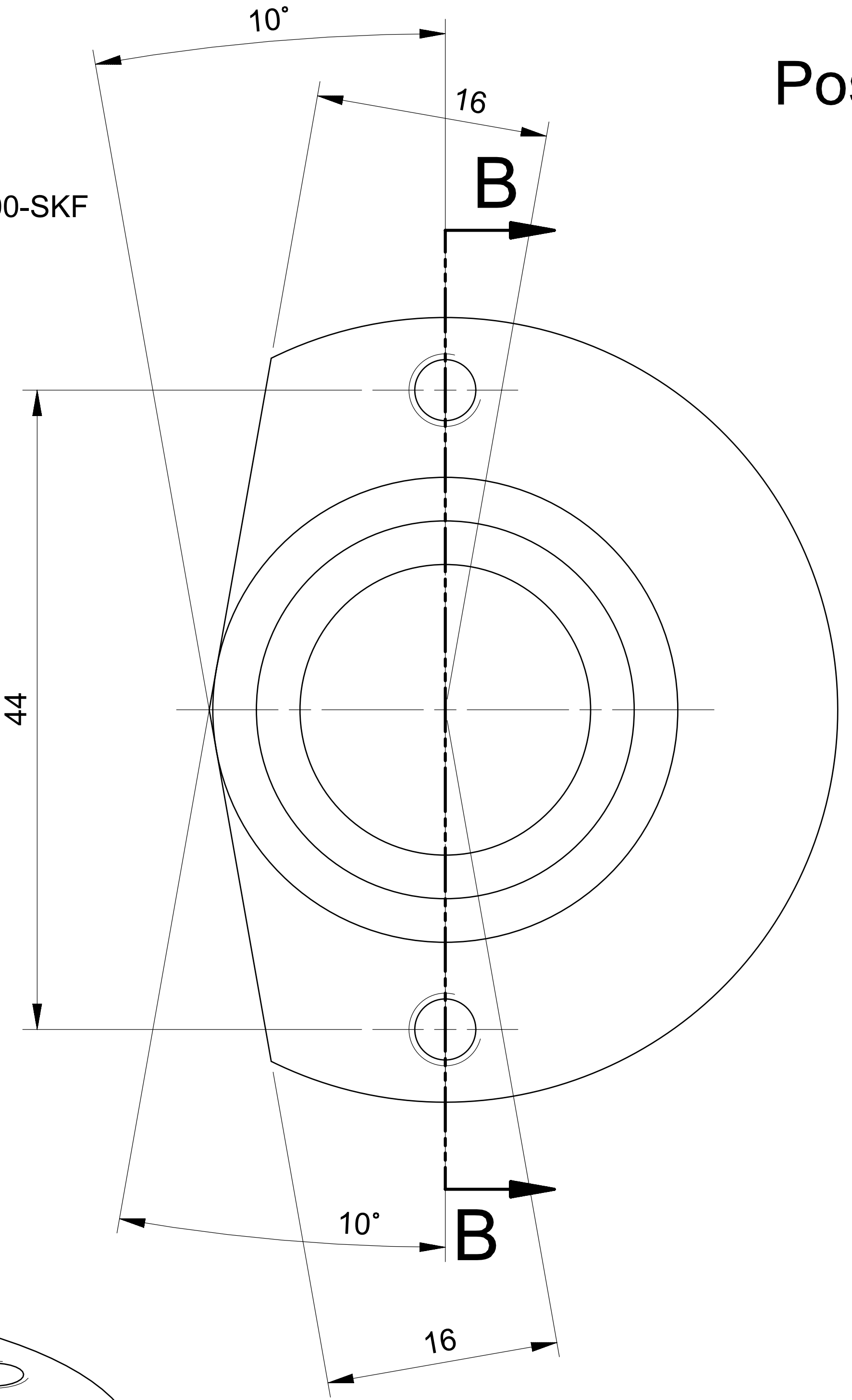

Pos. 1 (2:1) Alu

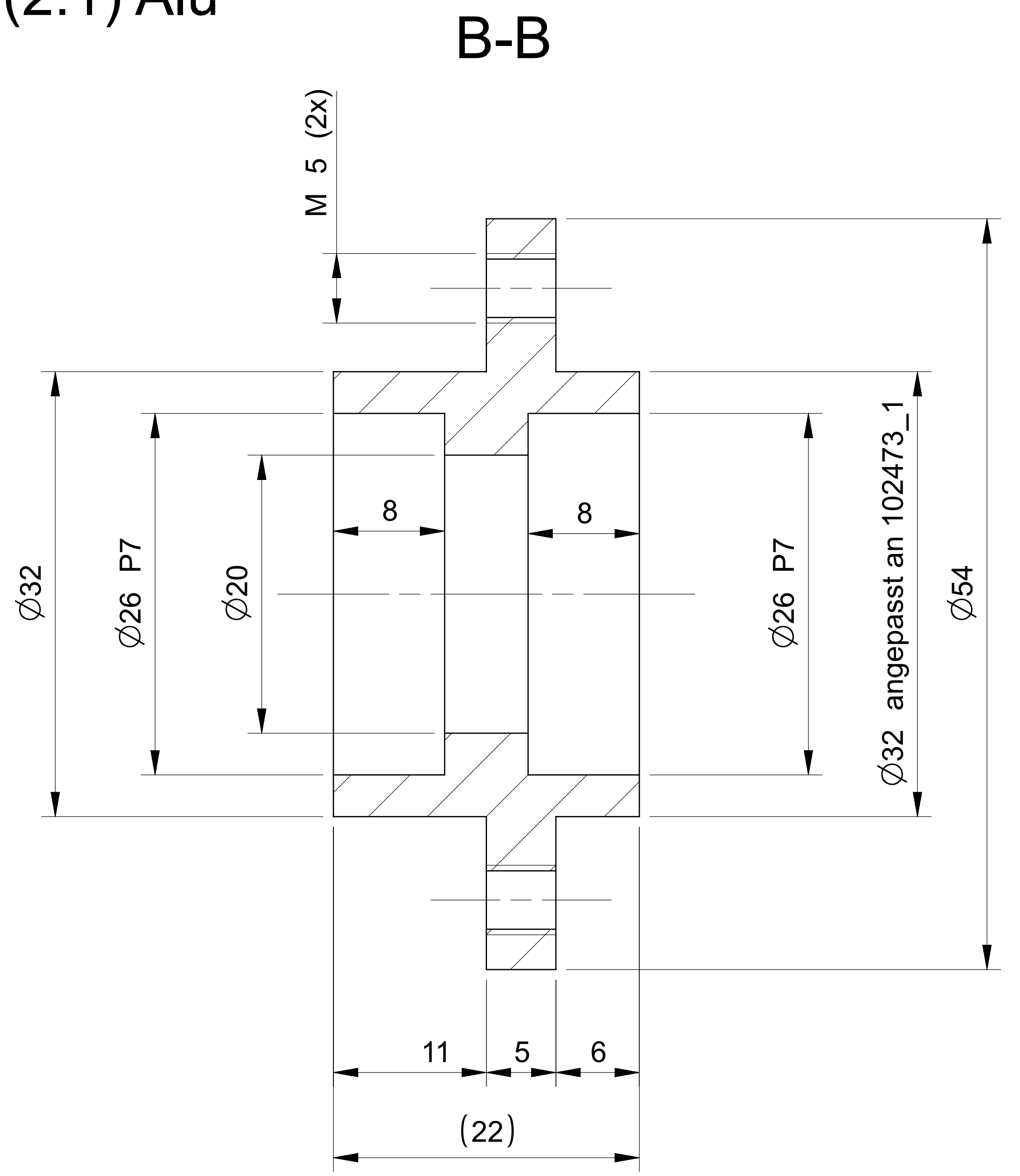

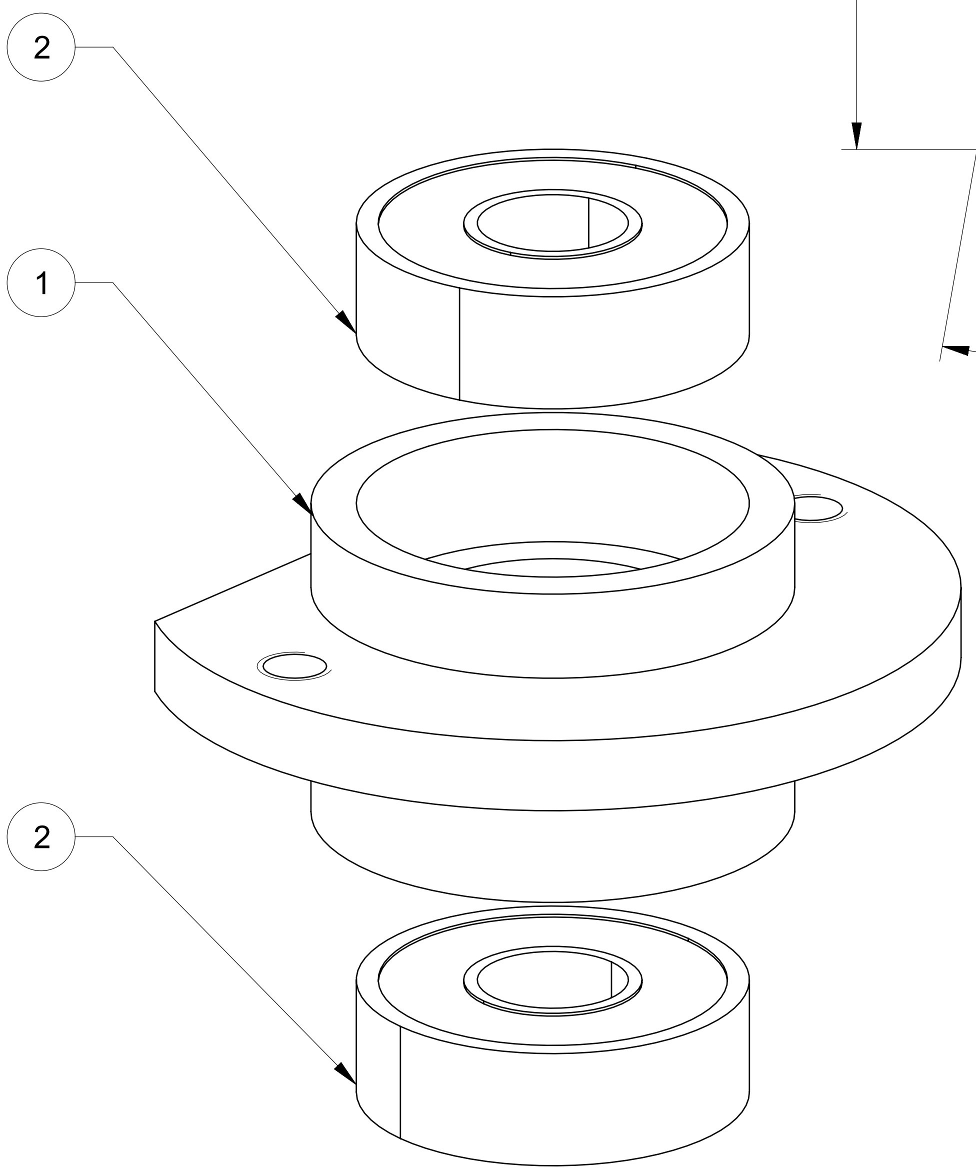

Allgemeintolleranzen ISO 2768-m

Pos Nr.	Anzahl	BENENNUNG	Teilenummer
2	2	Rillenkugellager 6000-SKF	500683_PRT
1	1	Lagerung	102476_1

Index	Datum	Name	Änderungen

Werkstoff	Ersatz für
Gewicht	Ersetzt durch

Benennung	Massstab		Datum	Name
Lagerung V2	1:1	Gezeichnet	10.01.2018	Collet
Landschaftsoekologie Insektenfalle		Geprüft		
		Freigeg,		

	Format	Zeichnungs-Nr.	Blatt
SLF WSL-Institut für Schnee- und Lawinenforschung SLF	A3	102476	1/1
