# Supplementary material for: Automated flight-interception traps for interval sampling of insects
Source: PLoS One. 2020 Jul 10;15(7):e0229476. doi: 10.1371/journal.pone.0229476 (PMC7351151; doi:10.1371/journal.pone.0229476)
Supplement: S7 Appendix — (ZIP) [file pone.0229476.s007.zip › AppendixG - Mechanical parts/pdf/102477.pdf]

Pos. 2 (2:1) Alu

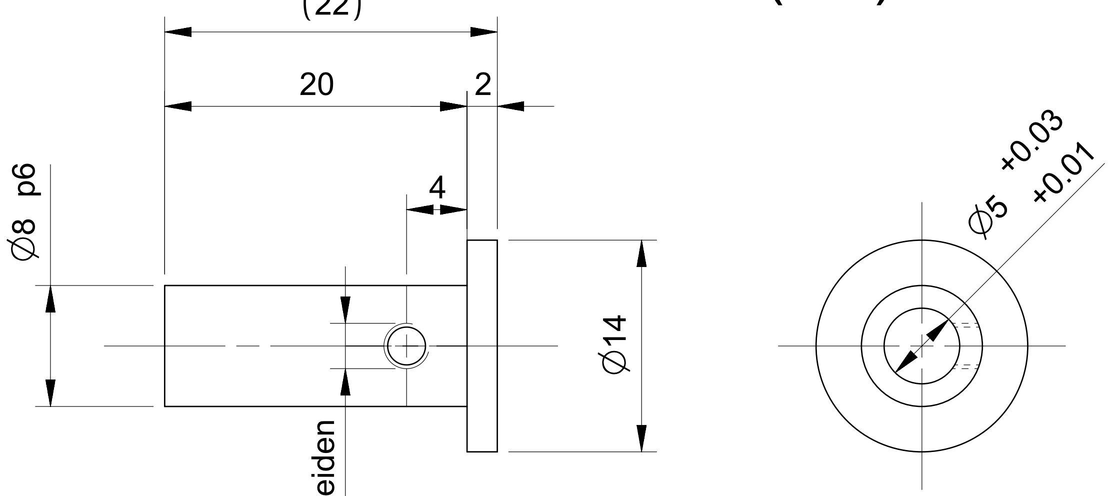

M 3 Gewinde mit Pos. 3 zusammen schneiden

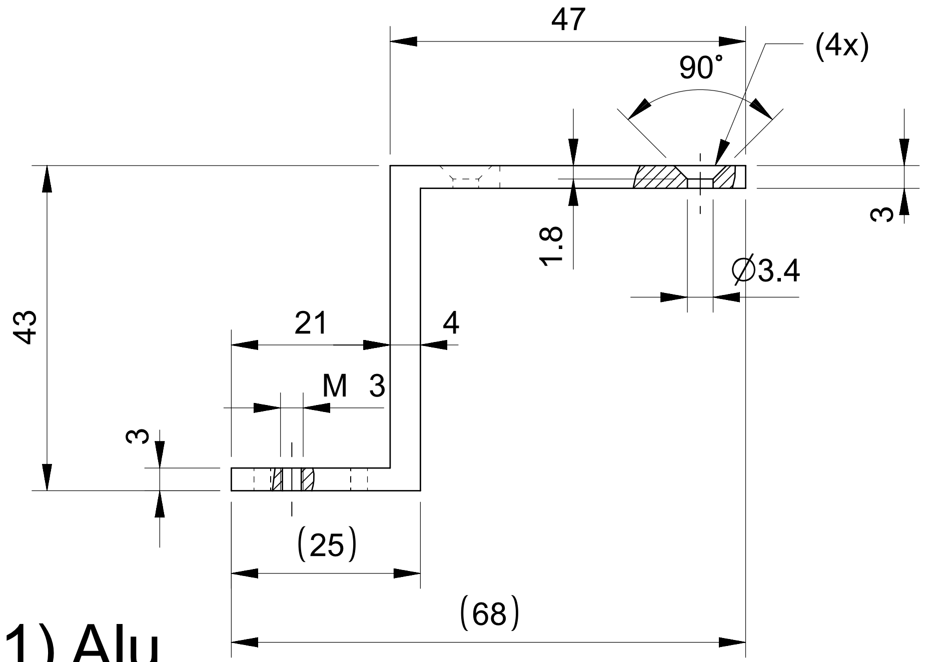

Pos. 1 (1:1) Alu

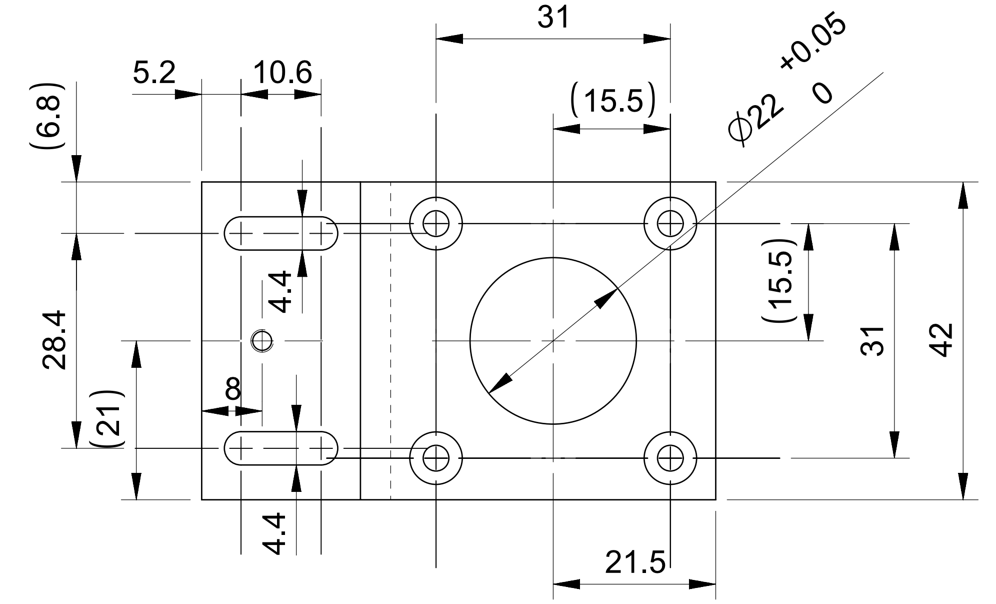

Pos. 3 (1:1) Pom-C

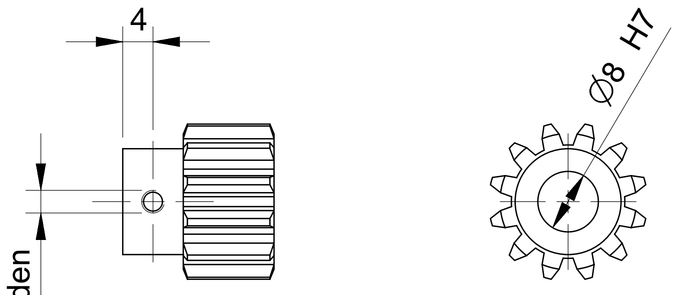

M 3 Gewinde mit Pos. 2 zusammen schneiden

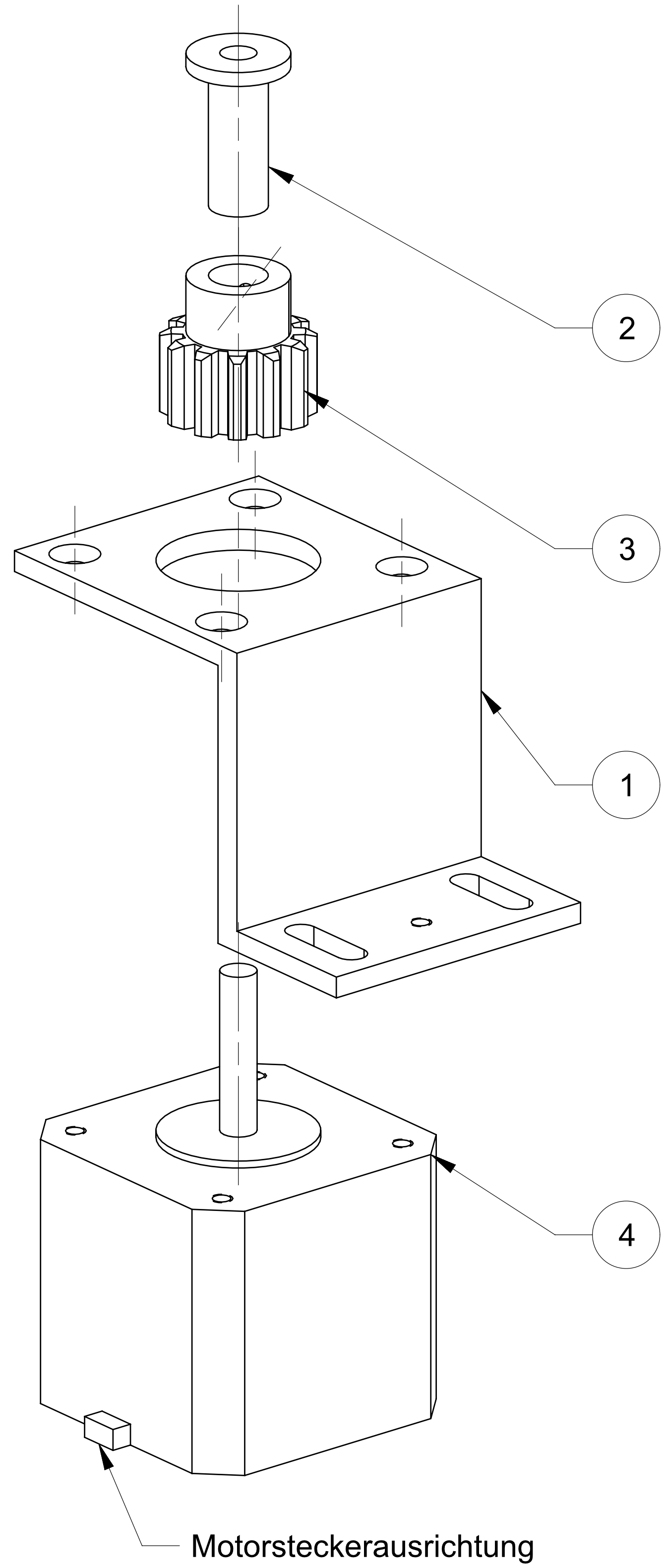

500684
Stirnzahnrad Polyacetal m1,5 12Z 21Dmm ;
Mädler; Artikelnummer 28501200

500685
Schrittmotor NEMA 17 Bipolar 200 Steps 42x38 2.8V 1.7A;
Pololu; Artikelnummer SY42STH38-1684A

Pos. 2 in Pos. 3 pressen, dann Gewinde M3 schneiden

Pos Nr.	Anzahl	BENENNUNG	Teilenummer
4	1	Schrittmotor NEMA 17	500685_PRT
3	1	Stirnzahnrad Polyacetal m1,5 12Z 21Dmm	500684_PRT
2	1	Huelse	102477_2
1	1	Winkel	102477_1

Index	Datum	Name	Änderungen

Werkstoff Alu / Pom-C		Ersatz für	
Gewicht		Ersetzt durch	

Benennung	Massstab		Datum	Name
Motoreinheit V2	1:1	Gezeichnet	04.02.2019	Collet
Landschaftsoekologie Insektenfalle		Geprüft	04.02.2019	
		Freigeg,		

	Format	Zeichnungs-Nr.	Blatt
SLF WSL-Institut für Schnee- und Lawinenforschung SLF	A3	102477	1/1
