# Supplementary material for: Automated flight-interception traps for interval sampling of insects
Source: PLoS One. 2020 Jul 10;15(7):e0229476. doi: 10.1371/journal.pone.0229476 (PMC7351151; doi:10.1371/journal.pone.0229476)
Supplement: S7 Appendix — (ZIP) [file pone.0229476.s007.zip › AppendixG - Mechanical parts/pdf/102473_22.pdf]

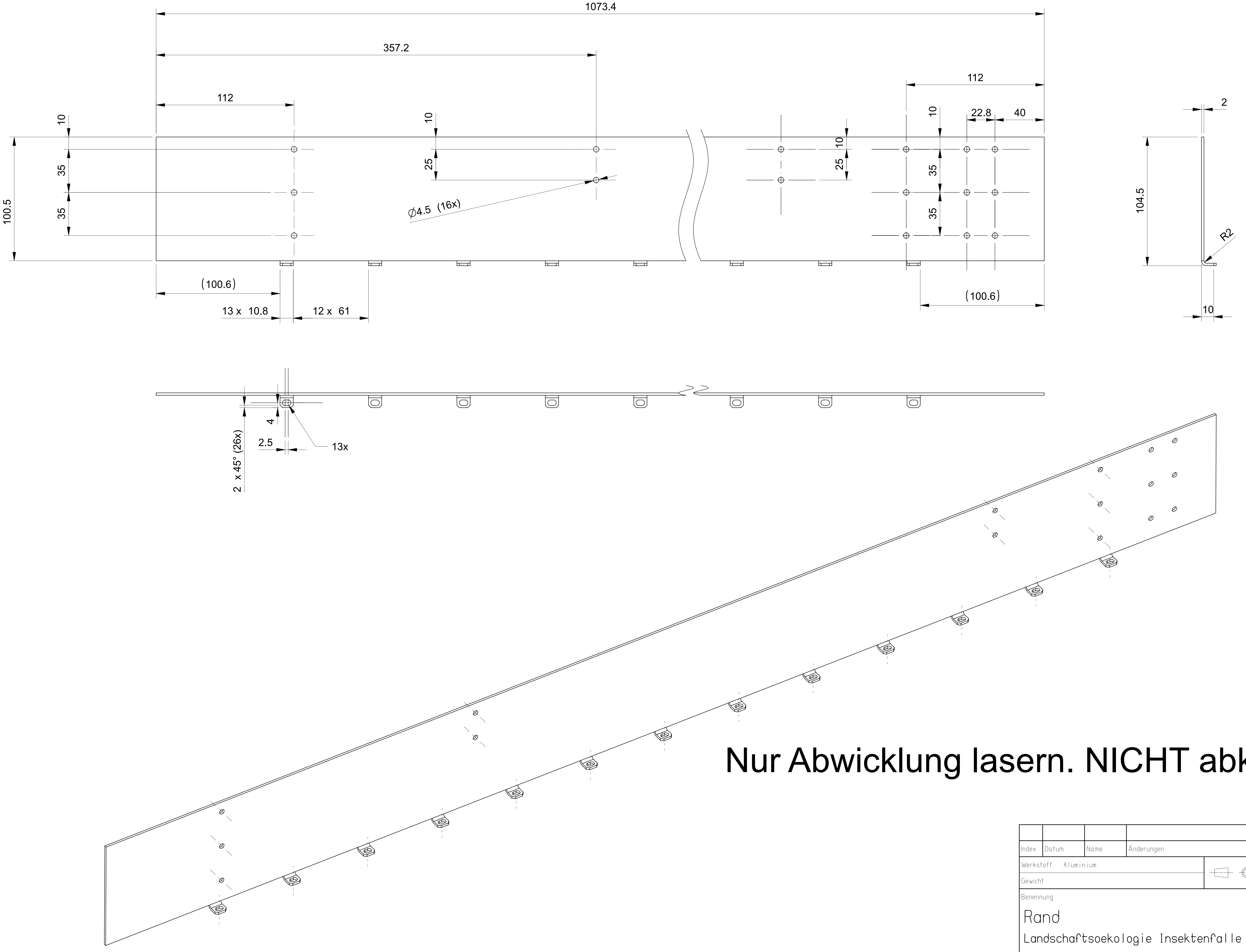

Nur Abwicklung lasern. NICHT abkanten!

Index	Datum	Name	Änderungen		
Werkstoff Aluminium				Ersatz für	
Gewicht				Ersatz durch	
Benennung	Massstab			Datum	Name
Rand	1:2	Gezeichnet		23.11.2018	Collet
Landschaftsoekologie Insektenfalle		Geprüft			
		Freigeg,			
WSL-Institut für Schnee- und Lawinenforschung SLF	Format A2	Zeichnungs-Nr. 102473_22_2			Blatt 1/1
